# Digital patient safety interventions in primary care: a systematic review and meta-analysis

**DOI:** 10.1186/s12916-026-05048-8

**Published:** 2026-08-03

**Authors:** Jung Yin Tsang, Claire Planner, Chloe-Nicole Low, Stuart Stewart, Charlotte Morris, Brandon Wong, Spyridon Marios Delitheos, George Kulangara, Lap Kan Derek Yu, Ashwanth Bosswell Suresh Babu, Alyaa Ajabnoor, Darren M Ashcroft, Maria Panagioti

**Affiliations:** 1 National Institute for Health and Care Research (NIHR) School for Primary Care Research, Centre for Primary Care and Health Services Research, School of Health Sciences, The University of Manchester, Manchester, UK; 2 National Institute for Health and Care Research (NIHR) Greater Manchester Patient Safety Research Collaboration (GMPSRC), The University of Manchester, Manchester, UK; 3 School of Medicine, University of Nottingham, Nottingham, UK; 4 Donal O’Donoghue Renal Research Centre, Salford Royal Hospital, Northern Care Alliance NHS Foundation Trust, Manchester, UK; 5 West London NHS Trust, London, UK; 6 Korgialeneio–Benakeio Hellenic Red Cross Hospital, Athens, Greece; 7 Lancashire Teaching Hospitals NHS Foundation Trust, Lancashire, UK; 8 Nottingham University Hospitals NHS Trust, Nottingham, UK; 9 Department of Pharmacy Practice, Faculty of Pharmacy, King Abdulaziz University, Jeddah, Saudi Arabia; 10 Division of Pharmacy and Optometry, School of Health Sciences, The University of Manchester, Manchester, UK

**Keywords:** Patient safety, Primary health care, Digital health, Clinical decision support systems, Electronic health records, Health information technology, Medical informatics applications, Medication errors, Delivery of health care

## Abstract

**Background:**

Patient safety events are among the top ten causes of death and disability worldwide, with most considered preventable, particularly in primary care. Digital interventions are increasingly used in primary care to improve patient safety, targeting errors and preventing adverse events. Evaluating their effectiveness and implementation is essential to inform clinical practice and policy. The study aims included a systematic review and meta-analysis of the effectiveness of digital patient safety interventions in primary care, comparing single and multicomponent interventions, identifying effective components, and exploring factors influencing their success.

**Methods:**

MEDLINE, EMBASE, and CENTRAL were searched from January 2001 to June 2025, restricted to English-language publications. This review followed PRISMA guidelines. Randomized controlled trials assessing digital interventions aimed at improving patient safety outcomes in primary care were included. Safety outcomes were grouped into medication safety and non-medication process measures, and adverse events. Two reviewers independently extracted and verified data and assessed risk of bias, resolving discrepancies by consensus. Random-effects meta-analysis was performed for predefined safety outcomes, and a narrative synthesis described implementation factors.

**Results:**

Seventy-four randomized controlled trials (RCTs) were included. Meta-analysis demonstrated benefits for medication safety process measures (k = 54; Odds Ratio (OR) 1.46, 95% confidence interval (CI): 1.30–1.64), non-medication process measures (k = 40; OR 1.77, 95% CI: 1.56–2.01), and adverse events (k = 12; OR 1.17, 95% CI: 1.06–1.30). Overall, multicomponent interventions consistently showed greater improvements than single-component interventions. Interventions combining components of clinical decision support and audit and feedback were most effective for medication safety, while patient-facing components had greater impact on non-medication process measures. Results remained consistent in sensitivity analyses restricted to higher-quality RCTs. Effectiveness was likely influenced by implementation factors, including workflow integration and relational support.

**Conclusions:**

Digital interventions in primary care are associated with moderate improvements across multiple patient safety process measures. Multicomponent approaches appear most promising, with effectiveness potentially shaped by implementation, patient engagement and contextual factors. These findings support the broader adoption of well-designed, contextually adapted digital strategies to enhance patient safety and underscore the need for ongoing evaluation of the sociotechnical factors supporting delivery of these interventions.

**Supplementary Information:**

The online version contains supplementary material available at 10.1186/s12916-026-05048-8.

## Background

Improving patient safety by reducing preventable patient harm is a global priority [1]. Patient safety incidents remain one of the top 10 causes of death and disability, costing healthcare systems several trillion dollars annually [1, 2]. Primary care delivers more than 80% of healthcare services worldwide, underscoring its pivotal role in preventing harm as nations work towards achieving universal health coverage [3]. Despite this, patient safety research has predominantly centered on in-hospital care reflecting perceptions of higher acuity and risk of harm in such settings [4]. It is estimated that 2–3% of consultations in primary care are linked to preventable patient safety incidents, most commonly involving medication related problems, diagnostic errors and delayed referrals [4–6]. There remains a pressing need to identify effective approaches to prevent patient harm and enhance the safety of primary healthcare systems.

Digital health interventions, which apply information and communication technologies to improve health and healthcare, are increasingly recognized for their potential to improve patient safety [7]. Their scalability, automation and reliability have been leveraged to enhance care quality and safety through mechanisms such as electronic health record (EHR) based tools, clinical decision support systems (CDSS) and audit and feedback (A&F), with some improvements in medication management and guidelines adherence [8–10]. However, measuring safety outcomes within these contexts is challenging, with many studies relying on process measures rather than measuring actual adverse events [11, 12]. For instance, about 1% of medication errors result in actual adverse drug events [13]. Although many safety outcomes have traditionally been easier to measure in secondary care, improvements in both digital infrastructure and primary care interventions now present enhanced opportunities for measurement and monitoring [4]. Yet, the evidence base concerning digital interventions targeting patient safety outcomes in primary care remains fragmented. It is also unclear whether multicomponent digital interventions, which integrate several digital functions, offer greater benefit compared with single-component approaches.

Therefore, we conducted a systematic review and meta-analysis to examine the effectiveness of digital interventions in improving patient safety outcomes in primary care, to assess differences between multicomponent and single-component interventions, to identify which specific components are effective, and to explore the factors contributing to their successes and failures.

## Methods

This study was reported in line with PRISMA standards for systematic reviews [4]. The study protocol was published on the International Prospective Register of Systematic Reviews (PROSPERO CRD42024620160).

### Search strategy and study selection

The searches were performed in MEDLINE, EMBASE and CENTRAL databases using Ovid^®^ from Jan 2001 up to Jun 2025. Search queries for English-language articles were developed focusing on four key areas: digital tools; patient safety; primary care; intervention studies. Full details of the search strategy and screening guide are detailed in Additional file 1: Table S1. All citations were imported into Covidence software for screening and data extraction [14]. Each paper was screened for inclusion by two independent reviewers at each stage, with disagreements being resolved via discussion with the wider review team. Eligible studies were randomized clinical trials (RCTs) with a direct comparator group (such as usual care, enhanced usual care or other active intervention), evaluating digital health interventions aimed at improving patient safety outcomes in primary care (see full eligibility criteria in Additional file 1: Table S2). A digital intervention was defined as incorporating at least one of the following elements: automation; actions performed at scale; meaningful improvements in the speed of communication (i.e., beyond simple email or text messages); computerized storage, searching, or retrieval of data providing an advantage over paper-based or offline equivalents; or the use of sensor data from digital devices with no analogue counterpart. Safety outcomes were grouped into three main categories based on prior patient safety studies, including medication safety process measures (inappropriate prescribing, appropriate prescribing for high-risk groups, medication errors); non-medication process measures (diagnostic errors or delays, timely investigations or laboratory monitoring, optimized management of high-risk groups); and adverse events (unplanned and cause specific hospitalizations, cardiovascular events, asthma exacerbations and adverse drug events) [4, 15]. 

### Data extraction

Data were extracted and checked by at least two independent reviewers using a screening guide and standardized data extraction tool (Additional file 1: Table S3). Extracted data included: country, primary care setting, study design, patient safety outcomes measured, patient safety outcome category, intervention characteristics, target population, sample size, and results including characteristics or implementation processes that contributed to successes or failures. For each digital intervention, data were extracted on whether it included one or more of four main components (CDSS; A&F; reminders; patient facing component) based on previous studies and the scope of included papers, with interventions being defined as single-component or multicomponent (i.e. more than one concurrent component) based on these [10, 16]. 

### Assessment of study quality

Risk of bias was assessed by two reviewers using the Cochrane Risk of Bias (RoB 2) tool for randomized trials, including the adapted version for cluster-RCTs [17]. This was completed concurrently with data extraction, with disagreements being resolved via discussion with the wider team.

### Statistical analysis

Meta-analysis was performed to examine the effect on each of the three categories of patient safety outcomes specified above. Random effects models were used in all analyses as we anticipated high heterogeneity from digital interventions [18]. Meta-analysis data was extracted by and verified by two independent authors (MP, JT). Most studies reported outcomes as event counts, with odds ratios (ORs) calculated in Stata version 14 using inverse-variance weighting, with study weights automatically calculated within Stata. When reporting formats differed, data were transformed and ORs derived using Comprehensive Meta-Analysis (CMA) software. Each patient safety outcome category was analyzed separately. Within each outcome category, three subgroup analyses were performed: comparing single-component versus multicomponent interventions (the primary analysis); interventions including CDSS, A&F, or both; and interventions with or without a patient-facing component. A sensitivity analysis was also conducted, whereby only studies with low risk of bias were retained in the analyses to examine the robustness of the results. For cluster RCTs, where studies reported adjusted effect estimates that accounted for clustering, these were extracted and used in the analyses. Where such adjustments were not available and intracluster correlation coefficients (ICCs) were not reported, published data were used as reported, which may introduce potential unit-of-analysis limitations. In studies with multiple intervention groups, the sample size and number of events in the shared control group were proportionally divided across intervention arms to prevent double-counting. Heterogeneity was quantified by using the I^2^ statistic with I^2^ values of 25%, 50%, and 75% indicating low, moderate, and high heterogeneity, respectively [19]. We inspected the symmetry of the funnel plots and used Egger’s test to examine for publication bias [20]. To supplement our findings of the meta-analysis, a descriptive narrative synthesis summarized included trials and examined intervention characteristics and implementation.

## Results

### Study characteristics

**Fig. 1:**
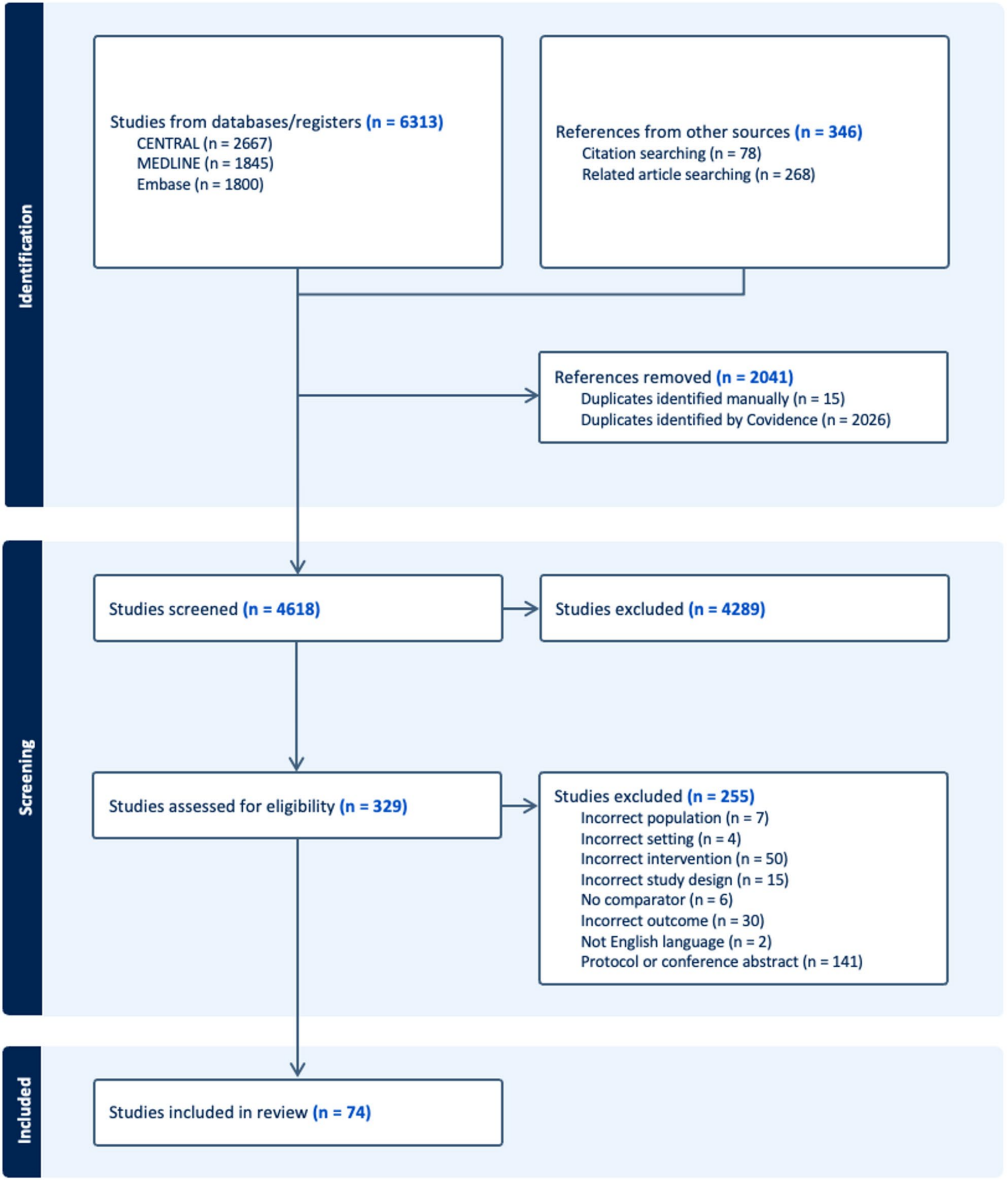
PRISMA flowchart. Flowchart of the selection process and numbers of studies included in the review

The searches identified 6313 citations in total. After 2041 duplicates were removed, 4289 citations were excluded from title and abstract screening. Of the 329 full texts reviewed, 74 were included in the review [21–94]. Fig. 1 shows more detail on study flow and selection process, with further details of study characteristics in Additional file 1: Table S4. The included RCTs covered 23 years in total, spanning from 2002 to 2025. Of the 74 RCTs, 58 (78.4%) were cluster RCTs, with 7 (9.5%) incorporating a stepped-wedge design. Among the included studies, 41 (55.4%) were from the USA or Canada, 13 (17.6%) were from the UK or Ireland, 8 (10.8%) from other European countries, 5 (6.8%) were from African countries, 4 (5.4%) from Australia, 2 (2.7%) from China, and 1 (1.4%) from Brazil. Participants across studies included a mix of healthcare professionals (including physicians, nurses, pharmacists, physician assistants and community health workers), populations (including adults and children), and patients with specific conditions (e.g. cardiovascular disease or urinary tract infections). Settings included primary care clinics and primary care outreach services.

### Intervention characteristics

Across the 74 RCTs, the most frequent intervention component was CDSS (*n* = 59, 79.7%), followed by reminders (*n* = 39, 52.7%), A&F (*n* = 34, 45.9%), and patient-facing components (*n* = 20, 27.0%). Overall, 55 RCTs (74.3%) included a multicomponent intervention (Table 1). Interventions targeted a wide range of clinical areas including general prescribing (*n* = 34, 45.9%), cardiovascular disease (*n* = 22, 29.7%), pediatric presentations (*n* = 6, 8.1%), cancer (*n* = 5, 6.8%), metabolic health (*n* = 4, 5.4%), and respiratory disease (*n* = 3, 4.1%). The mean duration of the intervention period was 13.8 months, ranging from 1 to 48 months. The most frequently reported patient safety outcome category was medication safety process measures in 42 RCTs (56.7%), with non-medication process measures were addressed in 30 RCTs (40.5%), and adverse events reported in 12 RCTs (17.6%). Comparator groups were diverse across the studies, including usual care being described in 53 RCTs (71.6%); additional education, information or training in 7 RCTs (9.5%); paper equivalents in 4 RCTs (5.4%); other comparator groups in 3 RCTs (4.1%); with comparator details not reported in 7 RCTs (9.5%). Further details on outcomes and intervention components are shown in Additional file 1: Table S5.

**Table 1:** Summary of intervention characteristics

		Count (%)
		Interventions may fall in multiple categories
**Intervention components**		
Clinical decision support system		59 (79.7%)
Audit and feedback		34 (45.9%)
Reminders		39 (52.7%)
Patient facing components		20 (27.0%)
Multicomponent (>1 of the above 4 components concurrently)		55 (74.3%)
**Specificity**		
Condition specific		47 (63.5%)
Drug specific		35 (47.2%)
Patient type	Adults only	56 (75.7%)
	Adults and Children	12 (16.2%)
	Children only	6 (8.1%)
**Safety outcome category**		
Medication safety process measures	Inappropriate prescribing	25 (33.8%)
	Appropriate prescribing for high-risk groups	15 (20.3%)
	Medication errors	2 (2.7%)
	*Total*	*42 (56.7%)*
Non-medication process measures	Timely investigations or laboratory monitoring	13 (17.6%)
	Diagnostic errors or delays	9 (12.2%)
	Optimized management of high-risk groups	8 (10.8%)
	*Total*	*30 (40.5%)*
Adverse events	Hospitalizations (unplanned or cause-specific)	7 (9.5%)
	Cardiovascular events	2 (2.7%)
	Asthma exacerbations	1 (1.4%)
	Adverse drug event	2 (2.7%)
	*Total*	*12 (16.2%)*
**Main clinical area**		
General prescribing (e.g. psychotropic medications, antibiotics, medication discrepancy)		34 (45.9%)
Cardiovascular disease (e.g. hypertension, diabetes, atrial fibrillation)		22 (29.7%)
Respiratory disease (e.g. asthma and chronic obstructive pulmonary disease)		3 (4.1%)
Metabolic health (e.g. liver disease, bone health)		4 (5.4%)
Cancer (mostly multiple sites, e.g., lung, prostate, breast)		5 (6.8%)
Pediatric presentations (e.g. acutely unwell children, respiratory tract infections)		6 (8.1%)

### Study quality and risk of bias

Of the 74 included studies, 21 (28.3%) were assessed as having a low risk of bias, 43 (58.1%) were judged to have some concerns, and 10 (13.5%) were considered at high risk of bias (Additional file 1: Table S6). The main sources of bias arose from deviations from the intended interventions, with 31 studies (41.9%) at moderate risk and 6 studies (8.1%) at high risk, often related to implementation fidelity. A smaller number of studies were deemed high risk due to selection processes (*n* = 3, 4.1%) or attrition (*n* = 3, 4.1%).

### Meta-analysis results on medication safety process measures

**Fig. 2:**
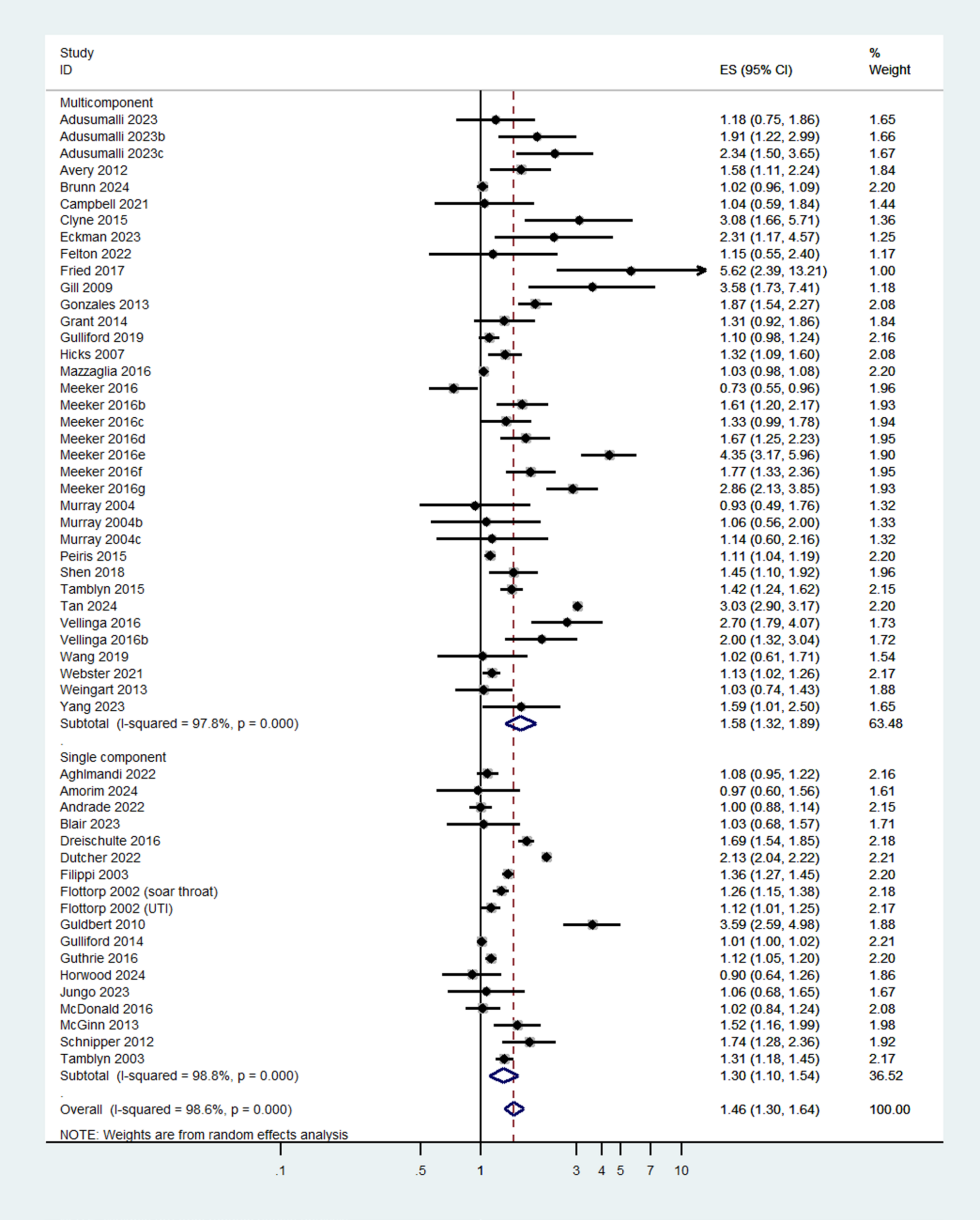
Meta-analysis of the effectiveness of digital interventions in improving medication safety process measures. The size of squares is proportional to the weight of each study, with the horizontal lines indicating the 95% confidence interval (CI) of each study; diamond = the pooled estimate with 95% CI; and ES = effect size

There were 42 RCTs, comprising 54 independent comparisons, which reported data on medication safety process measures. Across these comparisons (Fig 2), the pooled OR was 1.46 (95% CI: 1.30–1.64; I² = 98.6%), indicating a 46% greater likelihood of improvement in medication safety process measures compared with control groups (typically usual care). Egger’s test suggested potential small-study effects indicative of publication bias (intercept = 3.25; 95% CI: 0.80–5.70; *p* = 0.010), supported by visual inspection of the asymmetrical funnel plot (Additional file 1: Fig. S1). A sensitivity analysis including only the 17 studies assessed as having a low risk of bias (Additional file 1: Fig. S2) yielded a similar pooled OR of 1.27 (95% CI: 1.15–1.41; I² = 87.3%).

Multicomponent digital interventions, evaluated in 36 RCTs, showed an OR of 1.58 (95% CI: 1.32–1.89, I² = 97.8%) in medication safety process measures compared to usual care. The remaining 18 studies evaluating single-component digital interventions yielded a slightly lower OR of 1.30 (95% CI: 1.10–1.54, I² = 98.8%) compared to usual care. In analyses of specific digital intervention components (Additional file 1: Fig. S3 and Fig S4), interventions that combined CDSS with A&F (22 studies) were associated with the greatest improvement in medication safety process measures (OR = 1.75; 95% CI, 1.35–2.25), followed by A&F only interventions (7 studies; OR = 1.48; 95% CI, 1.10–1.99), and CDSS-only interventions (22 studies; OR = 1.21; 95% CI, 1.12–1.30). Interventions with a patient-facing component (13 studies) had OR = 1.41 (95% CI: 1.23–1.62), while non-patient-facing interventions (41 studies) had OR = 1.45 (95% CI: 1.26–1.67), indicating similar effects across these groups.

### Meta-analysis results on non-medication process measures

**Fig. 3:**
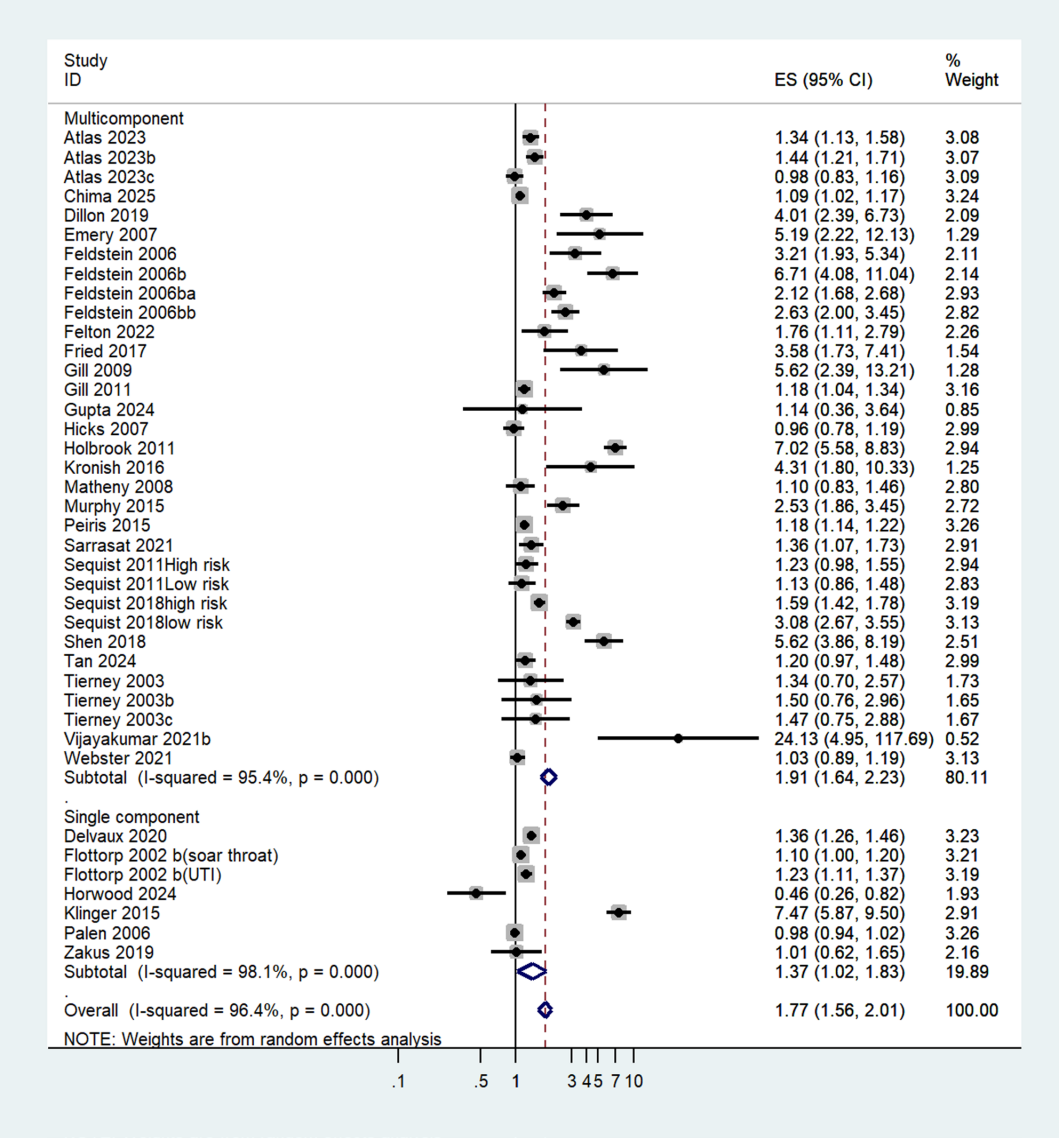
Meta-analysis of the effectiveness of digital interventions in improving non-medication related process measures. The size of squares is proportional to the weight of each study, with the horizontal lines indicating the 95% confidence interval (CI) of each study; diamond = the pooled estimate with 95% CI; and ES = effect size

Thirty RCTs, comprising 40 independent comparisons, reported data on non-medication process measures. Across these studies the pooled OR was 1.77 (95% CI: 1.56–2.01; I² = 96.4%), indicating a 77% greater likelihood of improving non-medication process measures compared with usual care (Fig 3). Egger’s test suggested potential small-study effects (intercept = 3.69; 95% CI: 1.72–5.67; *p* < 0.001), supported by some asymmetry of the funnel plot (Additional file 1: Fig. S5). A sensitivity analysis including only 12 studies assessed as having a low risk of bias (Additional file 1: Fig. S6) yielded a slightly lower, but still significant, pooled OR of 1.40 (95% CI: 1.15–1.69; I² = 96.5%). 

Multicomponent digital interventions, evaluated in 33 studies, demonstrated a 91% greater improvement (OR 1.91, 95% CI: 1.64–2.23, I² = 95.4%) in non-medication process measures compared with usual care. The single-component interventions, evaluated in 7 studies demonstrated 37% improvement (OR 1.37, 95% CI: 1.02–1.83, I² = 98.1%). In comparisons of specific digital components (Additional file 1: Fig. S7 and Fig S8), interventions combining CDSS with A&F (14 studies) demonstrated a 68% improvement (OR = 1.68, 95% CI: 1.40–2.01), similar to CDSS-only interventions (20 studies) that demonstrated a 90% improvement (OR = 1.70, 95% CI: 1.37–2.11). Interventions with a patient-facing component (13 studies) had a significantly larger effect (OR = 2.79, 95% CI: 1.97–3.95) than interventions without a patient-facing component (27 studies; OR = 1.37, 95% CI: 1.23–1.52).

### Meta-analysis results on adverse events

**Fig. 4:**
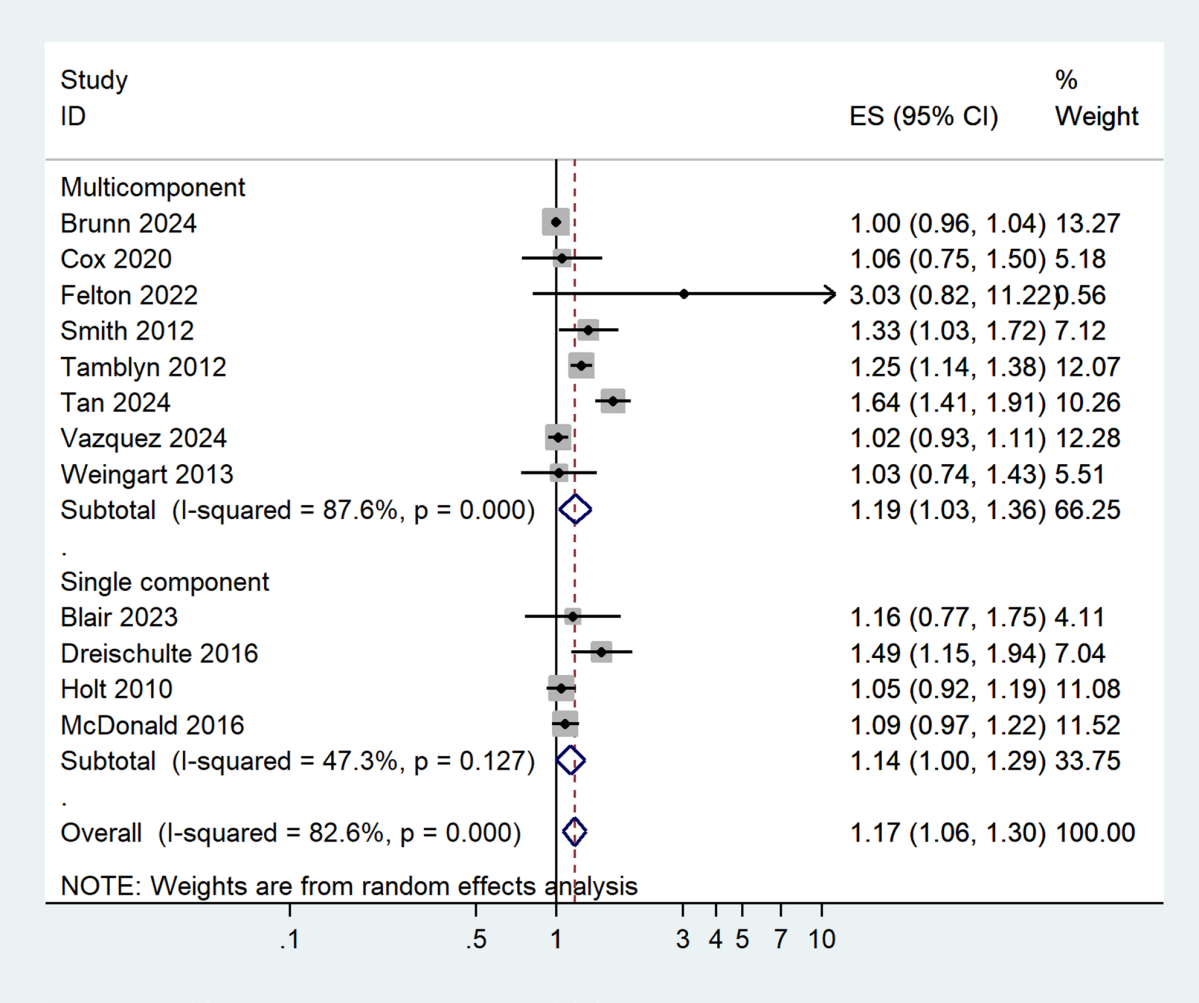
Meta-analysis of the effectiveness of digital interventions in reducing adverse events. The size of squares is proportional to the weight of each study, with the horizontal lines indicating the 95% confidence interval (CI) of each study; diamond = the pooled estimate with 95% CI; and ES = effect size

Twelve RCTs with 12 comparisons, reported data on the effects of digital interventions on adverse events. Across these studies, the pooled OR was 1.17 (95% CI: 1.06–1.30; I² = 82.6%), indicating a 17% greater likelihood of reducing adverse events compared with control groups (Fig. 4). Egger’s test suggested evidence of small-study bias (intercept = 2.08; 95% CI: 0.10–4.07; *p* = 0.04), consistent with visual inspection of the funnel plot (Additional file 1: Fig. S9). A sensitivity analysis including only the five studies assessed as having a low risk of bias (Additional file 1: Fig. S10) yielded a similar pooled OR of 1.18 (95% CI: 1.00–1.41; I² = 60.8%). 

Multicomponent digital interventions, evaluated in 8 RCTs, demonstrated a 19% reduction in adverse events (OR 1.19, 95% CI: 1.03–1.36; I² = 87.6%). Single-component digital interventions examined in 4 RCTs yielded a reduction of 14% (OR 1.14, 95% CI: 1.00–1.29; I² = 47.3%). Sample size was insufficient to compare individual digital components.

### Implementation factors and intervention characteristics contributing to variations in effects

Thirty studies (40.5%) described issues with implementation including complete lack of engagement, low usage of the digital eing utilized.^25–30,33,34,43,48–50,52,54,57,59,61,62,65,66,70,73–75,83−85,90,91,94^ Common barriers reported were difficulties integrating the intervention into existing systems and workflows, lack of time and resources, and diverse contextual issues (e.g. lack of wireless access in low resource settings). Multicomponent interventions often targeted multiple parts of workflow stages and decision points, with several (*n* = 13, 17.6%) reporting relational elements (e.g. academic detailing, pharmacists leading practice meetings) as critical for digital interventions to achieve organizational engagement, accountability and sustainability [26, 28, 29, 33, 34, 42, 49, 50, 52, 57, 61, 76, 83]. Twenty studies (27.0%) all incorporating CDSS components noted disparities between suggested actions and real-world clinical decision making (e.g. identifying a potential drug-interaction did not mean these risks outweighed benefits) [21, 23, 29, 46, 47, 49, 53, 57, 59, 67–69, 73, 74, 76, 77, 81, 82, 86, 90]. This was due to challenges in capturing complexity (e.g. changing guidelines, missing contraindications from free-text data) and developing actionable tailored responses, which were often too generic for both practitioners and patients. In contrast, nine studies (12.2%) reported that simpler interventions increased clinical relevance and actionability [23, 35, 36, 38–40, 58, 67, 82]. Five interventions (6.8%) with patient facing components also reported challenges in obtaining accurate and timely self-reported data [25, 46, 57, 61, 62]. 

## Discussion

### Summary of key findings

This systematic review demonstrates that digital interventions moderately improve patient safety outcomes in primary care. Benefits were observed across all safety domains, including both medication and non-medication process measures, along with mild reductions in adverse events. Multicomponent interventions were more effective than single-component interventions across outcomes, suggesting that combining strategies, such as CDSS, A&F, and patient-facing elements, enhances patient safety. The combination of CDSS and A&F appear particularly effective for medication safety process measures, whereas interventions with a patient-facing component appeared more influential for non-medication process measures. Despite these positive findings, there was substantial heterogeneity across studies and evidence of small-study effects across outcomes, which should be considered when interpreting the pooled estimates, particularly for adverse effects due to a smaller sample size. For implementation, interventions that used simple digital technology, that were meaningfully integrated into existing workflows, or were supported by relational elements (e.g., academic detailing or pharmacist-led meetings) tended to achieve greater engagement and impact. Further research is needed to understand the mechanisms and sociotechnical factors that enable digital health interventions to improve patient safety.

### Comparison with existing literature

To our knowledge, this is the largest meta-analysis that synthesizes the effects of digital health interventions across a range of patient safety outcomes in primary care. Our study provides broader evidence of the potential for digital interventions to improve patient safety in primary care and compares different digital intervention components. Previous reviews have examined the effects of CDSS and A&F interventions separately and in more specific clinical areas, such as medication safety, antibiotic prescribing, and cardiovascular risk reduction [95–101]. In general, these support that digital interventions have modest improvements in prescribing and clinician performance, but little impact on hospitalization or mortality [95–101]. In contrast, a recent meta-analysis focusing on medication safety suggests that organizational and structural interventions reduce hospital admissions, including multidisciplinary care models and system-level quality monitoring approaches, unlike professional education and CDSS alone [102]. This is consistent with the high heterogeneity observed in our effect sizes, and that multicomponent interventions were more effective. This critiques that single components may address too few aspects of decision-making and behavioral domains. Fundamentally, this highlights the importance of considering the sociotechnical aspects of how people work with technology in healthcare systems when designing digital interventions [103–107]. Nevertheless, long-term data remain limited, and clear evidence of sustained outcomes is lacking [104, 108]. There is increasing recognition that digital interventions operate within complex healthcare systems, where outcomes are shaped by interactions among technology, people, workflows, organizations, and the broader care environment [109]. This may explain our heterogeneous findings and underscores the need to design, implement, and evaluate digital interventions holistically rather than as isolated technological components.

### Limitations

This review captured a broad range of studies evaluating digital interventions in primary care across multiple countries and healthcare systems. Despite a systematic approach, we synthesized data from diverse geographical contexts, clinical domains and interventions, combining outcomes that may not be fully comparable across studies. Interventions varied in the extent of their digital components, even within subcategories such as clinical decision support systems, and many were multimodal, often including co-interventions, such as variable levels of education and financial incentives. As such, there was substantial heterogeneity across the studies which could overestimate the consistency of the observed effects, reflecting variations in intervention components, delivery formats, and primary care implementation contexts. The four main intervention components provided a high-level classification of digital interventions but may not have been sufficiently granular to capture all sources of heterogeneity. Future research should consider more detailed component-level analyses to better understand intervention effects once the field is more mature. Component network meta-analysis could be a promising approach for comparing digital intervention components; however, the current evidence base was not sufficiently developed due to limited replication of intervention types, few direct comparisons between digital interventions, and a predominance of usual care comparators, resulting in a sparse and inconsistent evidence base. The reminders intervention component showed substantial co-occurrence with CDSS and A&F components, and as such we did not conduct a separate subgroup analysis for this component. Some interventions fell outside our predefined scope, including telemonitoring, mental health apps, and handheld scanning devices. Our search was limited to RCTs within medical databases and English language articles, and the inclusion of wider studies and technology-focused databases (e.g., IEEE Xplore, ACM Digital Library) may have identified further relevant findings and reduced bias. The significant Egger’s test across outcomes indicates potential publication bias, but may also partly reflect heterogeneity from diverse interventions. While a narrative synthesis complemented our quantitative analysis, we did not examine qualitative studies or process evaluations, which could have provided more in-depth insights into implementation and contextual factors.

### Policy and practice implications

Our findings indicate that digital interventions can meaningfully enhance patient safety in primary care, supporting their wider integration into routine practice. Policymakers and healthcare leaders should prioritize interventions that are well-designed, contextually adaptable, and integrated into existing workflows, with attention to system usability and engagement strategies. Multicomponent approaches may offer additional promise by addressing multiple aspects of clinical decision-making, care processes, and patient engagement. Implementation strategies should also consider training, relational facilitation, and sociotechnical factors to maximize adoption, sustainability, and impact on patient-level outcomes.

## Conclusions

Digital interventions in primary care moderately improve patient safety outcomes, including both medication and non-medication process measures, along with modest reductions in adverse events. Effectiveness varied with implementation, context, and intervention design, with multicomponent interventions generally demonstrating greater impact. Future research should explore specific mechanisms and sociotechnical contexts enabling digital health interventions to improve patient safety.

## Supplementary Information

Below is the link to the electronic supplementary material.

Supplementary Material 1: Additional File 1: Supplementary content (Tables S1-S6 and Figures S1-S10).

## Data Availability

All data supporting the findings of this study are available within the paper and its Supplementary Information.

## Declarations

### Artificial intelligence (AI) use statement

Generative AI (ChatGPT 4.0) was used solely to assist with wording, grammar, and language refinement. All intellectual content, analysis, and conclusions were conceived, developed, and approved by the authors.

## Abbreviations

A&F: Audit and Feedback
CDSS: Clinical Decision Support System
CI: Confidence Intervals
CMA: Comprehensive Meta–Analysis
EHR: Electronic Health Records
ICC: Intracluster Correlation Coefficient
I²: I–squared statistic
OR: Odds Ratio
PRISMA: Preferred Reporting Items for Systematic Reviews and Meta–Analyses
PROSPERO: International Prospective Register of Systematic Reviews
RCT: Randomized Controlled Trial
RoB 2: Risk of Bias tool version 2
