# Supplementary material for: Digital patient safety interventions in primary care: a systematic review and meta-analysis
Source: BMC Med. 2026 Aug 3;24:407. doi: 10.1186/s12916-026-05048-8 (PMC13430711; doi:10.1186/s12916-026-05048-8)
Supplement: Supplementary file 1 — Supplementary Material 1: Additional File 1: Supplementary content (Tables S1-S6 and Figures S1-S10). [file 12916_2026_5048_MOESM1_ESM.docx]

**Additional file 1: Supplementary content**

Digital patient safety interventions in primary care: A systematic review and meta-analysis

**Contents**

[Table S1. Search strategies and terms 2](#_Toc232625963)

[Table S2. Screening Guide with inclusion and exclusion criteria 7](#_Toc232625964)

[Table S3. Data extraction tool 9](#_Toc232625965)

[Table S4. Summary of study characteristics 10](#_Toc232625966)

[Table S5. Details of intervention characteristics, components and outcomes 16](#_Toc232625967)

[Table S6. Risk of bias of included studies 23](#_Toc232625968)

[Figure S1. Visual funnel plot of meta-analysis on medication safety process measures 25](#_Toc232625969)

[Figure S2. Meta-analysis subanalysis on medication safety process measures including only 17 studies at low risk of bias 25](#_Toc232625970)

[Figure S3. Meta-analysis results on medication safety process measures comparing interventions including clinical decision support systems, audit and feedback, or both 26](#_Toc232625971)

[Figure S4. Meta-analysis results on medication safety process measures comparing interventions with or without a patient-facing component 27](#_Toc232625972)

[Figure S5. Visual funnel plot of meta-analysis on non-medication process measures 28](#_Toc232625973)

[Figure S6. Meta-analysis subanalysis on non-medication process measures including only 12 studies at low risk of bias 28](#_Toc232625974)

[Figure S7. Meta-analysis results on non-medication process measures comparing interventions including clinical decision support systems, audit and feedback, or both 29](#_Toc232625975)

[Figure S8. Meta-analysis results on non-medication process measures comparing interventions with or without a patient-facing component 30](#_Toc232625976)

[Figure S9. Visual funnel plot of meta-analysis on adverse events 31](#_Toc232625977)

[Figure S10. Meta-analysis subanalysis on adverse events including only 5 studies at low risk of bias 31](#_Toc232625978)

## Table S1. Search strategies and terms

| **Search for Ovid Medline**  1 Web Browser/  2 Patient Portals/  3 Online Systems/  4 Internet/  5 Cell Phone/  6 MP3-Player/  7 Computer Systems/  8 Mobile Applications/  9 Text Messaging/  10 Reminder Systems/  11 Speech Recognition Software/  12 Communications Media/  13 Telemedicine/  14 Telenursing/  15 (online* or web* or browser or portal or internet* or virtual*).mp.  16 ((cell* or mobile*) adj3 phone*).mp.  17 (handheld* or hand-held*).mp.  18 (smartphone* or smart-phone*).mp.  19 (personal* adj3 digital*).mp.  20 (PDA or "Palm OS" or "Palm Pre classic" or blackberry or nokia or symbian or INQ or HTC or sidekick or android* or iphone* or ipod* or ipad* or samsung or Huawei or sony or LG or pixel or (windows* adj3 (mobile* or phone*)) or (tablet adj3 (device* or comput*))).mp.  21 (app* adj3 (smartphone* or smart-phone or mobile* or phone* or tablet* or computer*)).mp.  22 (sms or mms).mp.  23 ((text* or short*) adj3 messag*).mp.  24 texting.mp.  25 ((electronic* or medication*) adj3 (reminder* or monitor* or record* or system* or device*)).mp.  26 (reminder adj3 (text* or system* or messag*)).mp.  27 alert*.mp.  28 wearable.mp.  29 ((interact* or speech* or voice* or touchtone) adj3 (recogni* or respon*)).mp.  30 IVR.mp.  31 (automat* adj3 (phone* or telephone* or call* or system*)).mp.  32 ("social media" or Facebook or Twitter or Instagram or Snapchat or YouTube or WhatsApp).mp.  33 (video* or television or radio or media* or multimedia or multi-media or audio* or webinar* or podcast* or wiki* or interactive or digital* or tech*).mp.  34 (telehealth* or tele-health* or telecare* or tele-care*).mp.  35 (mhealth or m-health or "m health" or "mobile health").mp.  36 (e-health or ehealth or "e health").mp.  37 or/1-36  38 Family Practice/  39 Primary Health Care/  40 Physicians, Family/  41 Community Health Services/  42 Community Dentistry/  43 Community Health Nursing/  44 Community Mental Health Services/  45 Community Pharmacy Services/  46 Home Care Services/  47 Community Mental Health Centers/  48 family pract$.tw.  49 general practice$.tw.  50 community based.tw.  51 community care.tw.  52 family medicine.tw.  53 family physician$.tw.  54 primary care.tw.  55 (primary health care or primary healthcare).tw.  56 family doctor$.tw.  57 primary medical care.tw.  58 general physician$.tw.  59 general practitioner$.tw.  60 primary care practitioner$.tw.  61 (community adj (health or healthcare or health care)).tw.  62 primary healthcare team$.tw.  63 primary health care team$.tw.  64 primary medical care team$.tw.  65 practice nurse$.tw.  66 practice manager$.tw.  67 (gpsi or gpwsi).tw.  68 (practitioner$ adj3 special interest$).tw.  69 (primary care or primary health care or general practice or family practice or family medicine).nw.  70 or/38-69  71 exp patient safety/  72 exp adverse outcome/  73 exp adverse drug reaction/  74 exp iatrogenic disease/  75 exp medical error/  76 exp malpractice/  77 patient safety.tw.  78 safety culture.tw.  79 (safe$ adj2 (practice$ or manage$)).tw.  80 iatrogenic disease$.tw.  81 malpractice$.tw.  82 (patient adj2 harm$).tw.  83 human error$.tw.  84 ((service$ or system$ or communication$ or organisation$ or organization$) adj2 (weak$ or fail$)).tw.  85 (latent adj1 (threat$ or cause$ or fail$)).tw.  86 ((adverse or avoidable or preventable or unsafe or safet$) adj2 (event$ or outcome$ or complication$ or death$ or effect$ or reaction$ or accident$ or injur$)).tw.  87 ((medica$ or diagnostic or therapeautic or administration or dispensing or prescri$) adj2 (error$ or mistake$ or fault$)).tw.  88 (patient$ adj2 (risk$ or incident$ or accident$)).tw.  89 near miss$.tw.  90 never event$.tw.  91 untoward incident*.tw.  92 serious incident*.tw.  93 serious report* event*.tw.  94 ((Quality & safety in health care or International Journal for Quality in Health Care).jn. or (Qual Saf Health Care or IJQHC).ja.) and safe$2.mp.  95 polypharmacy/  96 polypharmacy.tw.  97 (patient adj1 satisf*).tw.  98 Quality of Health Care/ or quality of care.mp.  99 or/71-98  100 37 and 70 and 99  101 randomized controlled trial.pt.  102 controlled clinical trial.pt.  103 randomized.ab.  104 placebo.ab.  105 clinical trials as topic.sh.  106 randomly.ab.  107 trial.ti.  108 101 or 102 or 103 or 104 or 105 or 106 or 107  109 100 and 108 |
| --- |
| **Search for Ovid Embase**  1 General Practice/  2 exp Primary Health Care/  3 General Practitioner/  4 family pract$.tw.  5 general practice$.tw.  6 community care.tw.  7 family medicine.tw.  8 family physician$.tw.  9 primary care.tw.  10 (primary health care or primary healthcare).tw.  11 family doctor$.tw.  12 primary medical care.tw.  13 general physician$.tw.  14 general practitioner$.tw.  15 primary care practitioner$.tw.  16 primary healthcare team$.tw.  17 primary health care team$.tw.  18 primary medical care team$.tw.  19 (gpsi or gpwsi).tw.  20 community pharmacy service$.tw.  21 (primary care or primary health care or general practice or family practice).jx.  22 ((general or family or primary or community or ambulatory) adj2 (care? or health$ or service?)).tw.  23 (GP? or ((general or family or primary or community or ambulatory) adj1 (practitioner* or physician* or doctor* or nurse* or provider*))).tw.  24 ((general or family or primary or community or ambulatory) adj1 (center* or centre* or practice*)).tw.  25 exp patient safety/  26 exp adverse outcome/  27 exp adverse drug reaction/  28 exp medical error/  29 exp malpractice/  30 patient safety.tw.  31 safety culture.tw.  32 (safe$ adj2 (practice$ or manage$)).tw.  33 iatrogenic disease$.tw.  34 malpractice$.tw.  35 (patient adj2 harm$).tw.  36 human error$.tw.  37 ((service$ or system$ or communication$ or organisation$ or organization$) adj2 (weak$ or fail$)).tw.  38 (latent adj1 (threat$ or cause$ or fail$)).tw.  39 ((medica$ or diagnostic or therapeautic or administration or dispensing or prescri$) adj2 (error$ or mistake$ or fault$)).tw.  40 (patient$ adj2 (risk$ or incident$ or accident$)).tw.  41 near miss$.tw.  42 never event$.tw.  43 untoward incident*.tw.  44 serious incident*.tw.  45 serious report* event*.tw.  46 polypharmacy/  47 polypharmacy.tw.  48 Web Browser/  49 Patient Portals/  50 Online Systems/  51 Internet/  52 Cell Phone/  53 MP3-Player/  54 Computer Systems/  55 Mobile Applications/  56 Text Messaging/  57 Reminder Systems/  58 Speech Recognition Software/  59 Communications Media/  60 Telemedicine/  61 Telenursing/  62 (online* or web* or browser or portal or internet* or virtual*).mp.  63 (handheld* or hand-held*).mp.  64 (smartphone* or smart-phone*).mp.  65 (sms or mms).mp.  66 ((text* or short*) adj3 messag*).mp.  67 texting.mp.  68 wearable.mp.  69 IVR.mp.  70 ("social media" or Facebook or Twitter or Instagram or Snapchat or YouTube or WhatsApp).mp.  71 (video* or television or radio or media* or multimedia or multi-media or audio* or webinar* or podcast* or wiki* or interactive or digital* or tech*).mp.  72 (telehealth* or tele-health* or telecare* or tele-care*).mp.  73 (mhealth or m-health or "m health" or "mobile health").mp.  74 (e-health or ehealth or "e health").mp.  75 ((cell* or mobile*) adj1 phone*).mp.  76 (personal* adj1 digital*).mp.  77 (PDA or "Palm OS" or "Palm Pre classic" or blackberry or nokia or symbian or INQ or HTC or sidekick or android* or iphone* or ipod* or ipad* or samsung or Huawei or sony or LG or pixel or (windows* adj3 (mobile* or phone*)) or (tablet adj1 (device* or comput*))).mp.  78 (app* adj1 (smartphone* or smart-phone or mobile* or phone* or tablet* or computer*)).mp.  79 ((text* or short*) adj1 messag*).mp.  80 ((electronic* or medication*) adj1 (reminder* or monitor* or record* or system* or device*)).mp.  81 ((interact* or speech* or voice* or touchtone) adj1 (recogni* or respon*)).mp.  82 (reminder adj1 (text* or system* or messag*)).mp.  83 (automat* adj1 (phone* or telephone* or call* or system*)).mp.  84 randomized.ab.  85 placebo.ab.  86 randomly.ab.  87 trial.ti.  88 controlled clinical trial/ or clinical trial/ or randomized controlled trial/ or "randomized controlled trial (topic)"/  89 or/1-24  90 or/25-47  91 or/48-83  92 or/84-87  93 89 and 90 and 91 and 92 |
| **Search for Ovid Central**  1 Web Browser/  2 Patient Portals/  3 Online Systems/  4 Internet/  5 Cell Phone/  6 MP3-Player/  7 Computer Systems/  8 Mobile Applications/  9 Text Messaging/  10 Reminder Systems/  11 Speech Recognition Software/  12 Communications Media/  13 Telemedicine/  14 Telenursing/  15 (online* or web* or browser or portal or internet* or virtual*).mp.  16 ((cell* or mobile*) adj3 phone*).mp.  17 (handheld* or hand-held*).mp.  18 (smartphone* or smart-phone*).mp.  19 (personal* adj3 digital*).mp.  20 (PDA or "Palm OS" or "Palm Pre classic" or blackberry or nokia or symbian or INQ or HTC or sidekick or android* or iphone* or ipod* or ipad* or samsung or Huawei or sony or LG or pixel or (windows* adj3 (mobile* or phone*)) or (tablet adj3 (device* or comput*))).mp.  21 (app* adj3 (smartphone* or smart-phone or mobile* or phone* or tablet* or computer*)).mp.  22 (sms or mms).mp.  23 ((text* or short*) adj3 messag*).mp.  24 texting.mp.  25 ((electronic* or medication*) adj3 (reminder* or monitor* or record* or system* or device*)).mp.  26 (reminder adj3 (text* or system* or messag*)).mp.  27 alert*.mp.  28 wearable.mp.  29 ((interact* or speech* or voice* or touchtone) adj3 (recogni* or respon*)).mp.  30 IVR.mp.  31 (automat* adj3 (phone* or telephone* or call* or system*)).mp.  32 ("social media" or Facebook or Twitter or Instagram or Snapchat or YouTube or WhatsApp).mp.  33 (video* or television or radio or media* or multimedia or multi-media or audio* or webinar* or podcast* or wiki* or interactive or digital* or tech*).mp.  34 (telehealth* or tele-health* or telecare* or tele-care*).mp.  35 (mhealth or m-health or "m health" or "mobile health").mp.  36 (e-health or ehealth or "e health").mp.  37 or/1-36  38 Family Practice/  39 Primary Health Care/  40 Physicians, Family/  41 Community Health Services/  42 Community Dentistry/  43 Community Health Nursing/  44 Community Mental Health Services/  45 Community Pharmacy Services/  46 Community Mental Health Centers/  47 family pract$.tw.  48 general practice$.tw.  49 community care.tw.  50 family medicine.tw.  51 family physician$.tw.  52 primary care.tw.  53 (primary health care or primary healthcare).tw.  54 family doctor$.tw.  55 primary medical care.tw.  56 general physician$.tw.  57 general practitioner$.tw.  58 primary care practitioner$.tw.  59 primary healthcare team$.tw.  60 primary health care team$.tw.  61 exp patient safety/  62 exp adverse drug reaction/  63 exp iatrogenic disease/  64 exp medical error/  65 exp malpractice/  66 patient safety.tw.  67 safety culture.tw.  68 (safe$ adj2 (practice$ or manage$)).tw.  69 iatrogenic disease$.tw.  70 malpractice$.tw.  71 (patient adj2 harm$).tw.  72 human error$.tw.  73 ((service$ or system$ or communication$ or organisation$ or organization$) adj2 (weak$ or fail$)).tw.  74 (latent adj1 (threat$ or cause$ or fail$)).tw.  75 ((adverse or avoidable or preventable or unsafe or safet$) adj2 (event$ or outcome$ or complication$ or death$ or effect$ or reaction$ or accident$ or injur$)).tw.  76 ((medica$ or diagnostic or therapeautic or administration or dispensing or prescri$) adj2 (error$ or mistake$ or fault$)).tw.  77 (patient$ adj2 (risk$ or incident$ or accident$)).tw.  78 near miss$.tw.  79 never event$.tw.  80 untoward incident*.tw.  81 serious incident*.tw.  82 polypharmacy/  83 polypharmacy.tw.  84 (patient adj1 satisf*).tw.  85 Quality of Health Care/ or quality of care.mp.  86 or/38-60  87 or/61-85  88 37 and 86 and 87 |

## Table S2. Screening Guide with inclusion and exclusion criteria

| **Screening guide – full text screening** *(version 2)*  Use the screening flowchart and additional guidance below to help you decide whether to select include or exclude.   \|  \|  \| \|  \| \| Is the setting Primary Care \| \| No \| EXCLUDE:  “wrong setting” \| \| \| --- \| --- \| --- \| --- \| --- \| --- \| --- \| --- \| --- \| --- \| \|  \|  \| \|  \| \|  \|  \|  \|  \| Yes \|  \|  \|  \|  \|  \| \|  \|  \|  \| \| Is it a ‘general population’ study? \| \| No \| \| EXCLUDE:  “wrong patient population” \| \| \|  \|  \| \|  \|  \| \|  \|  \|  \| Yes \|  \|  \|  \|  \|  \|  \| \|  \|  \|  \| Is the main component of the IV digital? \| \| No \| \| \| EXCLUDE:  “wrong intervention” \| \| \|  \|  \| \| \| \|  \|  \| Yes \|  \|  \|  \|  \|  \|  \|  \| \|  \| \| Are outcomes direct patient safety outcomes? \| \| No \| \| \| \| EXCLUDE:  “wrong outcomes” \| \| \|  \| \|  \| \| \| \| \|  \| Yes \|  \|  \|  \|  \|  \|  \|  \|  \| \|  \| Is the study using a randomised controlled trial design? \| \| No \| \| \| \| \| Exclude:  “wrong study design” \| \| \|  \|  \|  \|  \|  \|  \|  \|  \|  \|  \|   ‘**Include**’ is used when the article meets the criteria, in the circumstances outlined above  ‘**Exclude**’ should be used when the article does not meet one or more criteria  **Inclusion/exclusion criteria**  **Is the setting primary care?**   - A primary care setting includes general practice, dentistry, ophthalmology, or any other service commissioned by and delivered in primary care e.g. community mental health services, obstetrics etc. - The setting can be in a high income or low to middle income country - If there are multiple settings involved, such as in a transitional care intervention, the primary setting must be in primary care. If this is unclear **include** (and we will check with the study authors) - If the article meets all the other inclusion criteria but the setting is unclear **include** **& add a note** (and we will check with study authors)   **Is it a ‘general population’ study?**   - The intervention should be targeted at improving safety for the general population managed by primary care rather than disease specific populations typically managed by specialist services, such as specific cancers, complex mental health. - However, If the intervention is targeted at common conditions typically managed in primary care such as diabetes, hypertension, UTIs, then **include**   **Is the intervention a digital patient safety intervention**   - A digital intervention is defined as follows:   - where any part of the intervention includes automation   - where the digital element has improved the speed of communication in a way that is meaningful (e.g. an email or SMS being sent manually would not count unless the ability to receive the information quicker than by post was critical to the intervention)   - where the storage, searching or retrieval of data uses computerised means, and delivers a benefit over and above a paper or offline equivalent   - where actions are performed at scale   - where sensor data is used from digital devices which capture things with no analogue equivalent (e.g. GPS, accelerometer). Digital devices that replace an analogue equivalent (e.g. digital thermometer, digital weighing scales etc.) would not be included based on these criteria unless one of the previous 4 points was met. - Telehealth and/or telemonitoring interventions = exclude - If the mode of delivery is telephone = exclude - If the intervention is an educational intervention (without any additional digital component) delivered online = exclude   **Are outcomes direct patient safety outcomes?**   - We define a ‘direct patient safety outcome’ as: - Mortality - Hospitalization - Diagnostic error (missed, delayed, incorrect diagnosis) - Adverse drug reaction(s) - Inappropriate prescribing e.g. of antibiotics, polypharmacy, opioids / Optimised prescribing e.g. omission of indicated cardiovascular medicines - Medication error (in prescribing, dispensing, administering) - Failure to follow up (on test results, referrals, treatment) - Failure to carry out preventive care with high-risk populations   - - A direct patient safety outcome does not have to be the primary outcome.   - Examples of indirect patient safety outcomes are: - Interventions aimed at preventive care such as screening (in non-high-risk groups) - Quality of care outcomes such as compliance to checklists, population management of diabetes   **Is the study using a randomised controlled design**   - A randomised controlled design in which one group receives the digital intervention and one group does not, including cluster, stepped-wedge-randomised controlled trials. |
| --- | --- | --- | --- | --- | --- | --- | --- | --- | --- | --- | --- | --- | --- | --- | --- | --- | --- | --- | --- | --- | --- | --- | --- | --- | --- | --- | --- | --- | --- | --- | --- | --- | --- | --- | --- | --- | --- | --- | --- | --- | --- | --- | --- | --- | --- | --- | --- | --- | --- | --- | --- | --- | --- | --- | --- | --- | --- | --- | --- | --- | --- | --- | --- | --- | --- | --- | --- | --- | --- | --- | --- | --- | --- | --- | --- | --- | --- | --- | --- | --- | --- | --- | --- | --- | --- | --- | --- | --- | --- | --- | --- | --- | --- | --- | --- | --- | --- | --- | --- | --- | --- | --- | --- | --- | --- | --- | --- | --- | --- | --- | --- | --- | --- | --- | --- | --- | --- | --- | --- |

## Table S3. Data extraction tool

| \| Study ID \|  \| \| --- \| --- \| \| Year \|  \| \| Country \|  \| \| Participants (staff, patients, mix) with sample size (n) \|  \| \| Target population (with n) \|  \| \| Primary care setting (general practice, dentistry, other - specify) \|  \| \| Study design (RCT, cluster RCT, other controlled design) \|  \| \| Overall Aim/Objective \|  \| \| Outcomes reported of interest \|  \| \| Other outcomes reported (not to be extracted) \|  \| \| Direct patient safety outcome category \|  \| \| Brief description of intervention \|  \| \| Duration of intervention period \|  \| \| Control intervention \|  \| \| Follow up timepoints \|  \| \| Cost of intervention \|  \| \| Audit and feedback (0= no; 1= yes) \|  \| \| Decision support (0= no; 1= yes) \|  \| \| Reminders/ nudges (0= no; 1= yes) \|  \| \| Patient facing component (0= no; 1= yes) \|  \| \| Multicomponent (digital intervention has >1 component of 4 above) \|  \| \| Condition specific (0= no; 1= yes) \|  \| \| Drug specific (0= no; 1= yes) \|  \| \| Lab ordering or monitoring (0= no; 1= yes) \|  \| \| Child focused (0= no; 1= yes) \|  \| \| Risk of Bias (ROB) \|  \| \| Randomization Unit (Individual, PC practice, Team, Organisation, other] \|  \| \| Number (%) lost to follow up \|  \| \| ROB - randomization process/ selection bias \|  \| \| ROB - deviation from intended intervention/ classification \|  \| \| ROB - missing outcome data \|  \| \| ROB - measurement of outcome \|  \| \| ROB - selection of reported result \|  \| \| Overall ROB (High, some concerns, low, not reported) \|  \| \| Brief results/conclusions \|  \| \| Discussion on mechanisms of success (Characteristics/implementation?) \|  \| \| Discussion on mechanisms of failures/ limitations (Characteristics/implementation \|  \| |
| --- | --- | --- | --- | --- | --- | --- | --- | --- | --- | --- | --- | --- | --- | --- | --- | --- | --- | --- | --- | --- | --- | --- | --- | --- | --- | --- | --- | --- | --- | --- | --- | --- | --- | --- | --- | --- | --- | --- | --- | --- | --- | --- | --- | --- | --- | --- | --- | --- | --- | --- | --- | --- | --- | --- | --- | --- | --- | --- | --- | --- | --- | --- | --- | --- | --- | --- | --- | --- | --- | --- | --- | --- | --- | --- |

## Table S4. Summary of study characteristics

| **Study ID** | **Study Design** | **Country** | **Participants with sample size (n)** | **Target patient population and sample size (n)** | **Intervention Duration** |
| --- | --- | --- | --- | --- | --- |
| Adusumalli 2023 | RCT (cluster) | US | Primary care clinicians (n= 158) in n=28 practices | Patients not prescribed a statin (n=933) | 6 months |
| Aghlmandi 2023 | RCT (cluster) | US | Primary care physicians and pediatricians (n=3426) | Registered patients of all ages (n= 1,252,169) | 24 months |
| Amorim 2024 | RCT (cluster) | Brazil | GP Practices (n= 14) | Patients aged >= 60 years (n= 284) | 3 months |
| Andrade 2022 | RCT (cluster) | Australia | GPs (n= 2,552) | Veterans prescribed gabapentinoids and/or opioids (n= 3,271) | 2 weeks (3 x 1-day intereventions in 2 weeks) |
| Atlas 2023 | RCT (cluster) | US | Primary care practices (n= 44) | Patients with at least one overdue abnormal cancer screening test (n= 11,980) | 17 months |
| Avery 2012 | RCT (cluster) | UK | Primary care practices (n=72) | Registered patients of all ages (n= 480,942) | 6 months |
| Blair 2023 | RCT (cluster) | UK | Primary care practices (n= 144 intervention, n= 150 control); Staff included GPs, nurses, office staff, clinicians, pharmacists (n= 1339) | 0-9-year-old children (n= 336,496) | 12 months |
| Brunn 2024 | RCT (cluster, stepped wedge) | Germany | Primary care practices (n=688); Practitioners (GP and int med n=361) | Patients aged >= 18 with polypharmacy > 5+meds (n= 42,700) | 42 months |
| Campbell et al. 2021 | RCT (cluster) | US | Primary care practices (n= 10) | Adults aged 65 yo and older with an existing or new medication order for a target anticholinergic (n= 552) | 12 months |
| Chima 2025 | RCT (cluster) | Australia | GP Practices (n= 44) | Patients with abnormal blood tests (Anaemia +/- IDA, raised PLT, Raised PSA) associated with risk of undiagnosed cancer at baseline (n= 7555) | 12 months |
| Clyne 2015 | RCT (cluster) | Ireland | Primary care practices (n=21) | Patients aged >= 70 years with pre-existing potentially inappropriate prescribing (n=196) | 12 months |
| Clyne 2016 | RCT (cluster) | Ireland | Primary care practices (n= 21) | Patients aged >= 70 years with pre-existing potentially inappropriate prescribing (n=196) | 12 months |
| Cox 2018 | RCT (cluster) | Canada | Primary care providers (n=203) | Adult patients with AF (n= 1145) | 12 months |
| Cykert 2020 | RCT (cluster) | US | Primary care practices (n=219) | Patients aged 40-79 years with a calculated ASCVD 10-year risk score ≥10 at baseline (n=146,826) | 12 months |
| Delvaux 2020 | RCT (cluster) | Belgium | GPs (n= 272) | Patients (n=9683) with at least one of 17 indications (CVD management, hypertension, CKD, thyroid disease, type 2 diabetes mellitus, fatigue, anemia, liver disease, gout, ACS, lung embolism, rheumatoid arthritis, STI, acute diarrhea, chronic diarrhea, and follow-up of medication | 7 months |
| Dillon 2019 | RCT (cluster, stepped wedge) | UK | Primary care practices (n= 6) | Patients aged 18-75 with LFTs requested by their GP, excluding those with certain pre-existing conditions (control n= 490, intervention n= 64) | 6 months |
| Dreischulte 2016 | RCT (cluster, stepped wedge) | Scotland | Primary care practices (n=33); Patients (n=66,394) | Patients at risk of high-risk prescribing at one or more points in the preintervention period (n= 33, 334) and at one or more points in the intervention period (n= 33,060) | 48 weeks |
| Dutcher 2021 | RCT (cluster, stepped wedge) | US | Primary care providers (n= 183) across n= 30 primary care practices | Patients aged >= 18 who had a visit with a respiratory tract diagnosis (RTD) (n= 113,620) | 13 months |
| Eckman 2023 | RCT | US | Patients (n= 608) managed by primary care providers (n=187) | Patients with atrial fibrillation, receiving sub-optimal anticoagulation therapy, as determined by the Atrial Fibrillation Decision Support Tool (AFDST) (n= 608) | 23 months |
| Emery 2007 | RCT (cluster) | UK | 45 general practice teams with at least 3 physicians (n=135 minimum) | Patients concerned about familial breast/ovarian or colorectal cancer risk of those (n= 330 minimum) | 12 months |
| Feldstein 2006(A) | RCT (cluster) | US | Primary care providers (n= 200) across n=15 primary care practices | General adults receiving a new medication and had not received laboratory monitoring within 5 days after new dispensing (n= 1075) | 4 months |
| Feldstein 2006(B) | RCT | US | Primary care providers (n= 159) across n=15 primary care practices | Female patients aged 50 to 89 who suffered a fracture and had not received bone mineral density measurement or medication for osteoporosis (n=311) | 6 months |
| Felton 2022 | RCT | US | Physicians (n = 23) | Patients aged >= 45 (n=7190) | 3 months |
| Filippi 2003 | RCT (cluster) | Italy | GPs (n=300) | Patients with diabetes (n=20,626) | 7 months |
| Flottorp 2002 | RCT (cluster) | Norway | Primary care practices (n=142) | Patients with sore throat (n= 16,939) or with UTI (n= 9887) | 7 months |
| Fried 2017 | RCT | US | Patients (n= 156) at a Veteran Affairs Medical Centre (n=1) | Veterans aged >= 65 prescribed seven or more medications including at least one each for hypertension and diabetes mellitus (n= 156) | 90 days |
| Galt 2005 | RCT (cluster) | US | Primary care physicians (n= 78) across n= 31 primary care practices | General adult population (prescriptions evaluated n= 19,372 at baseline and n= 14,378 postintervention) | Not reported |
| Gill 2011 | RCT (cluster) | US | Clinicians (GPs and general medicine physicians, nurse practitioners, physician assistants) (n=119) | Patients (aged >= 65, taking NSAID not on gastroprotection, at risk of GI complications) (n= 5,234) | 12 months |
| Gill 2009 | RCT (cluster) | US | Primary care providers (n= 105) | Adult primary care patients aged 20–79 years (n=64,150) | 12 months |
| Gonzales 2013 | RCT (cluster) | US | Primary care practices in rural/ semi-rural areas (n=33) | Patients with acute bronchitis aged 13-64 (n=12, 776) | 6 months |
| Grant 2014 | RCT (cluster) | US | Primary care physicians (n= 44) | Patients with hypertension hyperlipidemia, type 2 diabetes (n= 3,655; intervention n= 2,049, control n= 1,606) | 12 months |
| Guldberg 2010 | RCT (cluster) | Denmark | GPs (n= 86) | Patients with T2DM (age 40-70) (n= 2,458) | 15 months |
| Gulliford 2014 | RCT (cluster) | UK | Primary care practices (n= 104) | All registered patients aged 18 to 59 years (n= 346,309) | 12 months |
| Gulliford 2019 | RCT (cluster) | UK | Primary care practices (n= 79) | Registered patients of all ages (n= 641,012) | 38 weeks |
| Gupta 2024 | RCT (cluster) | USA | Primary care providers (n=16) | Adult patients with anaemia and low ferritin (n=316) | 4 months |
| Guthrie 2016 | RCT (cluster) | Scotland | Primary care practices (n=262) | Registered patients particularly vulnerable to harm from the targeted prescribing at baseline (n= 170659) | 12 months |
| Hicks 2007 | RCT (cluster) | US | Primary care practices (n= 14) | Patients receiving hypertension care (n= 2, 027) | 19 months |
| Holbrook 2011 | RCT | Canada | Primary care practices (n= 49) | Patients aged >= 55 with risk factors for vasculopathy and/or history of vascular disease (intervention n=545, control n=557) | 7 months |
| Holt 2010 | RCT | UK | Patients (intervention n = 18912, control n = 19235) | Patients (n=38147) split into 4 groups: (1) known CVD or DM between ages 50-74 and CV risk >= 20% in 10 years (2) known CVD or DM between ages 50-74 with incomplete risk profile but assumed risk of >20 over 10 years (3) known CVD but not DM and not had BMs checked in last 3 years (4) not known to have CVD or DM and aged over 75 with persistently raised BP across 3 recent consecutive readings | 28 months |
| Horwood 2024 | RCT (cluster) | South Africa | Primary care clinic nurses (n=31) | Sick children attending the primary care clinic (n=291) | 5 months |
| Jungo 2023 | RCT (cluster) | Switzerland | GPs (intervention n = 21, control n = 22); Patients (intervention n = 160, control n = 163) | Elderly patients(>= 65) with polypharmacy (>= 5 medications for >= 90 days) and multi-morbid (>=3 chronic conditions on the basis of ICPC-2) (n = 323) | 12 months |
| Klinger 2015 | RCT (cluster) | US | Primary care practices (intervention n = 13, control n = 13); Patients (intervention n = 5143, control n = 5985) | Patients verified to have new starts for target medications; intervention (n=776), control (n = 776) | 22 months |
| Kronish 2015 | RCT (cluster) | US | GPs ( intervention n = 12, control n = 12) | Patients with uncontrolled hypertension (intervention n=65, control n=35) | 4 years |
| Mainous 2012 | RCT (cluster) | US | Primary care practices (intervention n= 9 , control n= 61) | Adult and paediatric patients with Acute Respiratory Infections ( n not reported) | baseline data collection Oct-Dec 2009; intervention and 15m follow up Jan 2010-March 2011. |
| Matheny 2008 | RCT (cluster) | US | Primary care physicians (n= 303) in ambulatory clinics (n=20) | Registered patients of all ages overdue for recommended laboratory monitoring (n= 1,922) | 6 months |
| Mazzaglia 2016 | RCT (cluster, open label) | Italy | GPs (n= 197) | Patients with high CVD risk ( n= 2120 patents with DM, n= 3956 with Acute MI , n= 2158 with stroke) | 24 months |
| McDonald 2016 | RCT (cluster) | US | Home health organisation nurses (n=500) | Home care patients with high medication regimen complexity (n=7919) | Not reported |
| McGinn 2013 | RCT (cluster) | US | Primary care providers (n= 168) | Registered patients attending the practice with pharyngitis or pneumonia during the study period (n= 984) | 2 weeks |
| Meeker 2016 | RCT (cluster) | US | Primary care providers (n 248) across n= 47 practices | General adult population aged >= 18 (n=14,753) | Not reported |
| Murphy 2015 | RCT (cluster) | US | Primary care providers (n= 72) | Patients with abnormal findings in the diagnostic evaluation of lung, colorectal and prostate cancer (n= 10, 673) | 15 months |
| Murray 2004 | RCT | US | Healthcare providers (general internists and internal medicine residents) (n= 150+) | Patients with uncomplicated hypertension (n= 712) | 28 months |
| Palen 2006 | Randomized prospective intervention study | US | Primary care practices (n= 16), Physicians (n= 207) | Patients receiving at least one of 25 study medications, included in Baseline Laboratory Monitoring Alerts Within a Computerized Physician  Order Entry (n= 26,586) | 12 months |
| Peiris 2015 | RCT (cluster) | Australia | Primary care practices (n= 60) | Patients (>=35yrs) at high risk of CVD (n= 38, 725) | 12 months |
| Sarrassat 2021 | RCT (cluster, stepped wedge) | Burkina Faso | Healthcare workers in n= 80 primary care facilities | Children aged between 2 months and 5 years old (n= 2724) | 39 months |
| Schnipper 2012 | RCT (cluster) | US | Primary care practices (n= 11) | Patients who submitted a medication “eJournal” (an intervention which allows patients to review and indicate updates to their medication lists, allergies, and if applicable, diabetes management information) prior to attending their upcoming primary care appointment (n= 541) | 18 months |
| Sequist 2011 | RCT | US | Mixed: Primary care clinicians (physicians, nurse practitioners, physician assistants) (n= 292) and patients (n= 7,083) | Adults ≥30 years old presenting with chest pain and no prior cardiovascular disease (n = 7,083) | 15 months |
| Sequist 2018 | RCT | US | Mixed: Primary care physicians (n= 153) and patients (n= 7, 691) | Adults aged 18–80 years with an established diagnosis of stage III CKD: High-risk patients (n = 3,947); Low-risk patients (n = 3,744) | 12 months |
| Shen 2018 | RCT | China | Village doctors (n=65) across n=24 village clinics | General population patients in rural China (n= 1048) | 12 months |
| Smith 2012 | RCT (cluster) | UK | Primary care practices (n= 29; intervention n=14, control n=15) | Patients aged >= 5 years at risk of severe asthma exacerbations (n= 911; intervention n=457, control n=454) | 12 months |
| Steinhubl 2018 | RCT and matched observational study of controls | US | Patients (n= 2659) | Patients at high risk of undiagnosed atrial fibrillation (n= 2659) | 12 months |
| Tamblyn 2012 | RCT (cluster, cross over) | Canada | GPs (n= 81) | Patients aged >= 65 with psychotropic medications (n= 5628) | 22 months |
| Tamblyn 2015 | RCT (cluster) | Canada | GPs (n= 81) | Asthma patients aged >= 5 (n= 4,447) | 33 months |
| Tamblyn2003 | RCT | Canada | GPs (n=107) | Patients aged >= 66 (n= 12, 560) | 13 months |
| Tan 2024(N) | RCT (cluster) | Tanzania | Primary care health facilities (n=40) | Children between 1 day old and 15 years old seeking care for acute medical or surgical conditions (n= 44,306) | 11months |
| Tan 2024(P) | RCT (cluster) | Tanzania | Primary care health facilities (n=40) | Children aged between 2 to 59 months old seeking care for acute medical or surgical conditions (n= 450) | 5 weeks |
| Tierney 2003 | RCT | US | GPs and Pharmacists (at 4 Primary care practices) | Patients with heart failure and/or ischaemic heart disease (n= 706) | 12 months |
| Vazquez 2024 | RCT (cluster) | USA | Primary care practices (n=141) | Adult patients with CKD, HTN and DM (n= 11821) | 12 months |
| Vellinga 2016 | RCT (cluster) | Ireland | GPs (n=71) | Patients coded for a UTI (n=3314) | 6 months |
| Vijayakumar 2021 | RCT | Norway | GPs (n=25) | Patients with newly diagnosed or established COPD (n=149) | Unclear (Next 5-10 patients seen) |
| Wang 2019 | RCT (cluster, stepped wedge) | US | Primary care providers (n= 14) | Patients with AF at high risk of stroke (CHADSVASC ≥2)(n=1727) | 14 months |
| Webster 2021 | RCT (cluster) | Australia | Primary care practices (n=71) | Patients using the HealthTracker app and identified to potentially " benefit from polypills" by the application (n=74,608) | 33 months |
| Weingart 2013 | RCT | US | Patients (n=738; 375 intervention, 363 control) | Patients receiving >= 1 prescription for a new medication | 3 months |
| Yang 2022 | RCT (cluster, open label) | China | Physicians (n=335) across n=79 rural primary care practices | General population (antibiotic prescriptions included n= 313,165) | 3 months |
| Zakus 2019 | RCT (cluster) | Niger | Community health workers (n=130) | Children (aged < 5) in a rural population (n= 520) presenting with diarrhea, malaria and pneumonia | 6 months |

## Table S5. Details of intervention characteristics, components and outcomes

| Study ID | Key clinical area | Sub-area | Patient safety outcome category | Summary | Audit and feedback | Decision support | Reminders/ nudges | Patient facing component | Condition specific | Drug specific | Child focused |
| --- | --- | --- | --- | --- | --- | --- | --- | --- | --- | --- | --- |
| Adusumalli 2023 | Cardiovascular disease | Lipid control - statin | Medication Safety | Clinical nudge providing prompt on electronic health record and monthly peer comparison feedback. Patient nudge sending text message reminding patient regarding appointments and statin therapy | ✔ | ✔ | ✔ | ✔ | ✔ | ✔ | ✘ |
| Aghlmandi 2023 | Antibiotics prescribing | - | Medication Safety | Quarterly antibiotics prescribing audit and feedback with peer benchmarking | ✔ | ✘ | ✘ | ✘ | ✘ | ✔ | ✘ |
| Amorim 2024 | General prescribing | Inappropriate prescribing | Medication Safety | Mobile application based on national guidelines which provides rationale for potential inappropriate prescriptions and suggest safer alternatives. | ✘ | ✔ | ✘ | ✘ | ✘ | ✔ | ✘ |
| Andrade 2022 | General prescribing | Gabapentin | Medication Safety | Postal intervention sending education materials to GPs, while a digital intervention delivers the same content via computer | ✔ | ✘ | ✔ | ✘ | ✘ | ✔ | ✘ |
| Atlas 2023 | Cancers | Multiple screening | Diagnostic error/delay | Electronic health record reminders, postal outreach letters and patient navigation phone calls to improve effectiveness of cancer screening follow-up | ✔ | ✔ | ✔ | ✔ | ✘ | ✘ | ✘ |
| Avery 2012 | General prescribing | Inappropriate prescribing | Medication Safety, Timely laboratory testing | Pharmacist-led electronic feedback system. Meeting with practice team after intervention to review medication errors and records | ✔ | ✔ | ✘ | ✘ | ✘ | ✘ | ✘ |
| Blair 2023 | Paediatrics presentations | Respiratory tract infections and antibiotics | Medication Safety, Adverse events | Digital risk stratification algorithm and prescribing guidance for children presenting with respiratory tract infections | ✔ | ✘ | ✘ | ✘ | ✔ | ✔ | ✔ |
| Brunn 2024 | General prescribing | Inappropriate prescribing | Medication Safety, Adverse events | A clinical decision support system alerting physicians to inappropriate prescriptions including interactions, contraindications, dosing errors and duplications. Physician could adjust medications and provide patients with a standardized medication plan | ✘ | ✔ | ✘ | ✔ | ✘ | ✘ | ✘ |
| Campbell et al. 2021 | General prescribing | Anti-cholinergic medications | Medication Safety | Intervention targeting tricyclic antidepressants and urinary antispasmodics. Customisable "Best practice alert" electronically notify providers of target anticholinergics, aligned with organisational priorities and suggest safer alternatives. Animated video on anticholinergic medication risk for patients. | ✘ | ✔ | ✔ | ✔ | ✘ | ✔ | ✘ |
| Chima 2025 | Cancers | - | Diagnostic error/delay | Computer software uses demographic and blood test data to identify patients require further follow up. Provides clinical decision support and recommendation according to national guidance. Benchmarking of practice included. | ✔ | ✔ | ✘ | ✘ | ✔ | ✘ | ✘ |
| Clyne 2015 | General prescribing | Inappropriate prescribing | Medication Safety | Three component intervention - Potentially inappropriate prescription review with a pharmacist with web-based treatment algorithm. GP then conduct medication reviews referring to the electronic treatment algorithm. Patient subsequently provided leaflet explaining potentially inappropriate prescribing and alternative treatment options | ✘ | ✔ | ✔ | ✘ | ✘ | ✘ | ✘ |
| Clyne 2016 | General prescribing | Inappropriate prescribing | Medication Safety | Three component intervention - Potentially inappropriate prescription review with a pharmacist with web-based treatment algorithm. GP then conduct medication reviews referring to the electronic treatment algorithm. Patient subsequently provided leaflet explaining potentially inappropriate prescribing and alternative treatment options | ✘ | ✔ | ✔ | ✘ | ✘ | ✘ | ✘ |
| Cox 2018 | Cardiovascular disease | Atrial fibrillation | Adverse events | Individualised, auto generated clinical decision support on atrial fibrillation care recommendations. It provided DOAC dosing support based on multiple factors and provide management suggestions | ✘ | ✔ | ✔ | ✔ | ✔ | ✘ | ✘ |
| Cykert 2020 | Cardiovascular disease | Medication optimisation | Medication Safety | Multi-component intervention including a Web-based education module and webinars, on-site practice facilitator who helps implementing changes, generation of a practice-specific cardiovascular population management dashboard. | ✔ | ✔ | ✘ | ✘ | ✔ | ✘ | ✘ |
| Delvaux 2020 | Multiple | Laboratory blood testing | Timely laboratory testing | Provides selection of laboratory test order sets according to the specific indications | ✘ | ✔ | ✘ | ✘ | ✔ | ✘ | ✘ |
| Dillon 2019 | Liver diseases | - | Diagnostic error/delay, Timely laboratory testing | An liver function testing system which sent electronic prompt for liver disease screening on abnormal liver function tests. After additional clinical data was entered, the system gave the choice of additional tests. | ✔ | ✔ | ✔ | ✘ | ✔ | ✘ | ✘ |
| Dreischulte 2016 | General prescribing | Inappropriate prescribing | Medication Safety, Adverse events | Web-based tool flagging high-risk prescriptions, prompting physicians to confirm/stop/adjusting medications | ✔ | ✘ | ✘ | ✘ | ✘ | ✔ | ✘ |
| Dutcher 2021 | Antibiotics prescribing | Respiratory tract infections | Medication Safety | An initial education session on appropriate prescribing for respiratory tract infections and patient communication strategies. Monthly electronic feedback is then given to physicians regarding their antibiotics prescribing performance | ✔ | ✘ | ✘ | ✘ | ✔ | ✔ | ✘ |
| Eckman 2024 | Cardiovascular disease | DOACs prescribing in Atrial fibrillation | Medication Safety | A Best practice advisory was added to the Atrial Fibrillation Decision Support Tool in electronic health record. It alerted clinicians when a patient could benefit from a change in anticoagulation therapy. | ✔ | ✔ | ✔ | ✘ | ✔ | ✔ | ✘ |
| Emery 2007 | Cancers | Breast, ovarian and colorectal cancers | Diagnostic error/delay, Appropriate management of high-risk group | Computer based decision support system for physicians provided tailored risk and management advice on family history of breast, ovarian and colorectal cancer. | ✘ | ✔ | ✘ | ✔ | ✔ | ✘ | ✘ |
| Feldstein 2006 (A) | Osteoporosis | Bisphosphonate and BMD measurements | Timely laboratory testing | Patient-specific electronic medical record messages included guideline resources on osteoporosis evaluation, calcium and vitamin D intake, lifestyle, and medications. A follow-up message was sent at 3 months if no bone mineral test or osteoporosis treatment had been ordered. | ✘ | ✔ | ✘ | ✔ | ✔ | ✔ | ✘ |
| Feldstein 2006 (B) | Osteoporosis | - | Timely laboratory testing | Three components to improve laboratory monitoring: Electronic medical record reminder notified clinicians if patient had not completed recommended lab test within required timeframe. Personalised automated voice message reminding patients about the required lab tests. Nurse and pharmacist outreach to encourage testing. | ✔ | ✘ | ✔ | ✘ | ✘ | ✔ | ✘ |
| Felton 2022 | Cardiovascular disease | Atrial Fibrillation | Medication Safety, Adverse events | Electronic risk alert which screens for risk of stroke and cardiovascular disease in line with newly developed guidelines. Physician can then use an order set to prescribe medications and reduce risk factors | ✘ | ✔ | ✔ | ✘ | ✔ | ✔ | ✘ |
| Filippi 2003 | Cardiovascular disease | Diabetic patients | Medication Safety | An electronic reminder when GPs opened the records of diabetic patients aged ≥30. It alerted them to potential high cardiovascular risk and suggested considering anti-platelet therapy. Function could be deactivated based on physician preference. | ✘ | ✘ | ✔ | ✘ | ✔ | ✔ | ✘ |
| Flottorp 2002 | Antibiotics prescribing | Urinary tract infections in female, Pharyngitis | Medication Safety, Timely laboratory testing | Multi-component intervention includes providing recommendations and patient education material in both electronic and poster format. Computer based decision support and reminders during consultation. Increased fee for telephone consultations. Printed material for primary care practice discussion and interactive training. | ✘ | ✔ | ✘ | ✘ | ✔ | ✘ | ✘ |
| Fried 2017 | General prescribing | Inappropriate prescribing | Medication Safety, appropriate management of high-risk group | Two-part web app: 1. Extracts medication and health data from electronic health record, 2. Supports medication reviews. It combines electronic health record data, chart reviews, and patient phone assessments to check for inappropriate medications, dosing issues, and side effects. Clinicians receive medication recommendations and patients get a report before appointments. | ✔ | ✔ | ✘ | ✔ | ✘ | ✘ | ✘ |
| Galt 2005 | General prescribing | Medication adverse effects | Medication Safety | Clinical drug information application on a personal digital assistant while prescribing. Physicians can enter and print prescriptions using the personal digital assistance. | ✔ | ✔ | ✘ | ✘ | ✘ | ✔ | ✘ |
| Gill 2011 | General prescribing | NSAIDs | Medication Safety, appropriate management of high-risk group | Electronic health record based clinical decision support which alerts when patient on an NSAIDs medication was deemed high risk. Prescribing tool provided to start or amend medications and tool to print patient education leaflets. Education materials also provided for physicians. | ✘ | ✔ | ✔ | ✘ | ✔ | ✔ | ✘ |
| Gill 2009 | Cardiovascular disease | Statins and lipid testing | Medication Safety, Timely laboratory testing | Interactive disease management tool embedded into the electronic medical record. A screening page alerted physicians if lipid testing is outstanding or goal not reached. Electronic assessment page displaying lipid targets and management page allowed medication adjustment, lab test ordering and printing education material | ✘ | ✔ | ✔ | ✘ | ✔ | ✔ | ✘ |
| Gonzales 2013 | Antibiotics prescribing | Respiratory tract infections | Medication Safety | Printed Decision Support via poster with acute cough algorithm in examination room and patient education leaflets. Computer based decision support generating recommended order sets for common tests and treatment options | ✘ | ✔ | ✔ | ✘ | ✔ | ✘ | ✘ |
| Grant 2014 | Cardiovascular disease | Hyperlipidaemia, Diabetes, Hypertension | Medication Safety, Timely laboratory testing | Integrated medication IT tool allowing future laboratory tests to be scheduled on prescribing medications for chronic conditions. The system tracked laboratory test and provided automatic reminders and outreach to patients | ✘ | ✔ | ✔ | ✔ | ✔ | ✔ | ✘ |
| Guldberg 2010 | General prescribing | Diabetic medications | Medication Safety | An electronic feedback system presented a list of Type 2 diabetes patients and physicians given option to use data during consultation or have an overview of the quality of their diabetes care with peer comparison. | ✔ | ✘ | ✘ | ✘ | ✔ | ✘ | ✘ |
| Gulliford 2014 | Antibiotics prescribing | Respiratory tract infections | Medication Safety | Decision support tool embedded in software which provides antibiotics prescribing guidelines | ✘ | ✔ | ✘ | ✘ | ✔ | ✔ | ✘ |
| Gulliford 2019 | Antibiotics prescribing | Respiratory tract infections | Medication Safety | Training webinar, automated monthly feedback reports of antibiotics prescribing and electronic support tools to inform appropriate prescribing | ✔ | ✔ | ✔ | ✘ | ✔ | ✔ | ✘ |
| Gupta 2024 | Cancers | Colorectal cancer | Timely investigation | Alert and decision support for patients with IDA, order set offered prioritised referral for endoscopy and gastroenterology consultation. | ✘ | ✔ | ✔ | ✘ | ✔ | ✘ | ✘ |
| Guthrie 2016 | General prescribing | Inappropriate prescribing | Medication Safety | Prescribing safety data feedback with or without theory information encouraging behaviour changes | ✔ | ✘ | ✘ | ✘ | ✘ | ✔ | ✘ |
| Hicks 2007 | Cardiovascular disease | Hypertension | Medication Safety, appropriate management of high-risk group | Clinical decision support tool integrated into electronic patient record, analyse patient data and identify patients with hypertension. It provided reminder to clinicians highlighting medications potential deviating from guidelines | ✘ | ✔ | ✔ | ✘ | ✔ | ✘ | ✘ |
| Holbrook 2011 | Cardiovascular disease | Diabetes | Appropriate management of high-risk group | Web based individualised vascular tracking and advice-decision support system tracked key vascular factors and diabetic risk factors. Colour coding helped prioritise risk factors | ✘ | ✔ | ✘ | ✔ | ✘ | ✘ | ✘ |
| Holt 2010 | Cardiovascular disease | Cardiovascular events | Adverse events | e-Nudge' system analyses electronic health record data using the Framingham risk equation to identify patients at cardiovascular risk. Physicians receive on-screen reminders about risk factors and missing data | ✘ | ✘ | ✔ | ✘ | ✔ | ✘ | ✘ |
| Horwood 2024 | Paediatrics presentations | Acutely unwell children - | Diagnostic error/delay | Electronic clinical decision support system receiving input from clinicians, provide classifications and treatment recommendations for sick children. | ✘ | ✔ | ✘ | ✘ | ✘ | ✘ | ✔ |
| Jungo 2023 | General prescribing | Medication optimisation | Medication Safety | Web-based decision support system using STOPP/START criteria to detect medication overuse, underuse and misuse. It provides recommendations by analysing medication, chronic conditions, lab data and observations. | ✘ | ✔ | ✘ | ✘ | ✘ | ✘ | ✘ |
| Klinger 2015 | General prescribing | Drug-related symptoms | Medication Safety | Interactive voice response technology to contact patient asking about potential drug-related symptoms. | ✘ | ✘ | ✘ | ✔ | ✘ | ✘ | ✘ |
| Kronish 2015 | Cardiovascular disease | Hypertension | Medication Safety | Electronic adherence report for eligible patients and clinical decision support providing suggestions on clinical actions | ✔ | ✔ | ✘ | ✔ | ✔ | ✔ | ✘ |
| Mainous 2012 | Antibiotics prescribing | Respiratory tract infections | Medication Safety | Clinical decision support tool integrating guidance and provide suggestion on medication prescription, considering presenting symptoms and patient characteristics | ✔ | ✔ | ✔ | ✘ | ✔ | ✘ | ✘ |
| Matheny 2008 | General prescribing | Medication monitoring | Timely laboratory testing | Electronic reminders delivered to primary care physicians about recommended laboratory testing | ✘ | ✔ | ✔ | ✘ | ✘ | ✔ | ✘ |
| Mazzaglia 2016 | Cardiovascular disease | Medication monitoring | Medication Safety | Clinical decision support tool which provides electronic reminder message triggered by patient characteristics and specific prescriptions. Electronic guidance and risk of medication interactions is then shown to clinician | ✘ | ✔ | ✔ | ✘ | ✔ | ✔ | ✘ |
| McDonald 2016 | General prescribing | Inappropriate prescribing, hospitalization | Medication Safety, Adverse events | Computerised algorithm identifying patients with significant polypharmacy and provide clinical decision support with alerts and recommendations | ✘ | ✔ | ✘ | ✘ | ✘ | ✘ | ✘ |
| McGinn 2013 | Antibiotics prescribing | Respiratory tract infections | Medication Safety | Integrated clinical prediction rules during patient encounters prompting clinician to complete a risk score calculator, providing management recommendations based on the score. | ✘ | ✔ | ✘ | ✘ | ✔ | ✔ | ✘ |
| Meeker 2016 | Antibiotics prescribing | Respiratory tract infections | Medication Safety | Three behavioural interventions were used alone or in combination to reduce inappropriate antibiotics prescribing. Suggestion on alternative non-antibiotic treatments, prompt for justification and peer comparison tool on inappropriate prescribing rate | ✔ | ✔ | ✘ | ✘ | ✘ | ✔ | ✘ |
| Murphy 2015 | Cancers | Lungs, Colorectal and Prostate cancers | Diagnostic error/delay | Electronic health record-based trigger algorithms to identify patients at risk of diagnostic delays | ✘ | ✔ | ✔ | ✘ | ✔ | ✘ | ✘ |
| Murray 2004 | Cardiovascular disease | Hypertension | Medication Safety, Hospitalisations | Computerised treatment suggestions for hypertension management to physicians and pharmacists using an electronic medical record system | ✘ | ✔ | ✔ | ✘ | ✔ | ✘ | ✘ |
| Palen 2006 | General prescribing | Medication monitoring | Timely laboratory testing | Computerised Provider Order Entry system providing non-intrusive laboratory test monitoring alerts | ✘ | ✘ | ✔ | ✘ | ✔ | ✘ | ✘ |
| Peiris 2015 | Cardiovascular disease | Blood pressure, statin monitoring | Timely investigations/ laboratory testing | Point of care electronic decision support, audit and feedback tool. The system also includes a web-based portal allowing health services to compare performance with peers | ✔ | ✔ | ✔ | ✔ | ✔ | ✔ | ✘ |
| Sarrassat 2021 | Paediatrics presentations | Acutely unwell children | Diagnostic error/delay | Electronic clinical decision support system on tablet computers providing recommendations on diagnosis, medication prescription, referral and counselling. | ✔ | ✔ | ✘ | ✘ | ✔ | ✔ | ✔ |
| Schnipper 2012 | General prescribing | Medication adherence | Medication Safety | Medication e-Journal allowing patient to highlight compliance to prescribed regime, report side effects and order repeat prescriptions. | ✘ | ✘ | ✘ | ✔ | ✘ | ✘ | ✘ |
| Sequist 2011 | Cardiovascular disease | Chest pain evaluation | Diagnostic error/delay, Medication Safety | Electronic health record alerts based on the Framingham Risk Score provided clinicians with investigation and treatment recommendations during consultations | ✔ | ✔ | ✔ | ✘ | ✔ | ✔ | ✘ |
| Sequist 2018 | Chronic kidney disease | - | Timely laboratory testing | Electronic health record alerts recommending nephrology referrals, medication prescriptions and laboratory tests. Patient engagement material mailed to promote self-management, providing tailored advice | ✔ | ✔ | ✔ | ✔ | ✔ | ✔ | ✘ |
| Shen 2018 | Antibiotics prescribing | - | Medication Safety, Timely investigations | Web-based intervention providing guidelines and information, with feedback on performance of individual clinician and percentages of antibiotic prescription | ✔ | ✔ | ✘ | ✔ | ✔ | ✔ | ✘ |
| Smith 2012 | Asthma | Asthma control & exacerbations | Adverse events | Asthma risk registers flagged at-risk patients in electronic health records with alerts visible to staff. Practices received a training session to improve engagement and asthma management using these alerts. | ✔ | ✔ | ✔ | ✘ | ✔ | ✘ | ✘ |
| Steinhubl 2018 | Cardiovascular disease | Atrial fibrillation | Diagnostic error/delay | Wearable ECG patch with continuously monitor heart tracing at home | ✘ | ✘ | ✘ | ✔ | ✔ | ✔ | ✘ |
| Tamblyn 2012 | General prescribing | Psychotropics medications | Medication Safety | A risk monitor alert based on prediction model shows a patient's injury risk score when a psychotropic drug was started or when patient's drug profile is opened. A visual thermometer showed new risk levels depended on medication adjustment. | ✘ | ✔ | ✔ | ✘ | ✘ | ✘ | ✘ |
| Tamblyn 2015 | Asthma | Asthma control | Medication Safety | Electronic alert shown to clinician for patients with suboptimal asthma control. Then a decision support can be used to automatically generate new prescription and advice on referral pathways for asthma home care | ✘ | ✔ | ✔ | ✘ | ✔ | ✘ | ✘ |
| Tamblyn2003 | General prescribing | Inappropriate prescribing, injury risk | Medication Safety, Adverse events | Prescribing system providing alert for specific known prescription problems with information and alternatives | ✘ | ✔ | ✘ | ✘ | ✘ | ✘ | ✘ |
| Tan 2024  (N) | Paediatrics presentations | Acutely unwell child, hospitalization | Medication Safety, Adverse events | Electronic clinical decision support algorithm which offers antibiotics prescription advice considering recent blood results and vitals. | ✔ | ✔ | ✔ | ✘ | ✔ | ✘ | ✔ |
| Tan 2024  (P) | Paediatrics presentations | Acutely unwell child, hospitalization | Medication Safety, Adverse events | Electronic clinical decision support algorithm which offers antibiotics prescription advice considering recent blood results and vitals. | ✔ | ✔ | ✔ | ✘ | ✔ | ✘ | ✔ |
| Tierney 2003 | Cardiovascular disease | Ischaemic heart disease, heart failure | Appropriate management of high-risk group | Computer based cardiac care order suggestion in medication list. Electronic referencing guidelines can be viewed by physician. Pharmacist would also receive alert on the care suggestions | ✘ | ✔ | ✔ | ✘ | ✔ | ✘ | ✘ |
| Vazquez 2024 | Cardiovascular disease | Hospitalization | Adverse events | Electronic health record-based clinical decision support which identifies patients with a combination of chronic kidney disease (CKD), Hypertension and Diabetes. | ✔ | ✔ | ✔ | ✘ | ✔ | ✔ | ✘ |
| Vellinga 2016 | Antibiotics prescribing | Urinary tract infections | Medication Safety | Digital information on national prescribing guidelines, reminder when Urinary tract infection coded onto electronic health system and offered audit of performance in practice | ✔ | ✔ | ✔ | ✔ | ✔ | ✔ | ✘ |
| Vijayakumar 2021 | COPD | COPD exacerbations and control | Appropriate management of high-risk group | Provide treatment advice and summary of COPD management information electronically. | ✘ | ✔ | ✘ | ✘ | ✔ | ✔ | ✘ |
| Wang 2019 | Cardiovascular disease | DOACs prescribing in Atrial fibrillation | Medication Safety | An algorithm that identifies patients on electronic health record who may benefit from starting a DOAC medication. Anticoagulation management service then received a list of this for review by pharmacist. | ✔ | ✔ | ✘ | ✘ | ✔ | ✔ | ✘ |
| Webster 2021 | Cardiovascular disease | Overall risk reduction, hypertension, Lipid control | Medication Safety, Appropriate management of high-risk group | Automated pop-up of tailored guideline-based recommendation according to individual cardiovascular risk factors. It then provided audit comparing performance of peer practices | ✔ | ✔ | ✔ | ✔ | ✔ | ✘ | ✘ |
| Weingart 2013 | General prescribing | Medication adverse effects | Medication Safety, Adverse drug events | Automated electronic message to patients highlighting potential medication issues and offer option to report side effects | ✘ | ✘ | ✔ | ✔ | ✘ | ✘ | ✘ |
| Yang 2022 | Antibiotics prescribing | - | Medication Safety | Health Information System-based pop-up warnings, a 10-day prescription summary feedback and the distribution of educational manuals | ✔ | ✔ | ✘ | ✘ | ✘ | ✘ | ✘ |
| Zakus 2019 | Paediatrics presentations | Acutely unwell children | Appropriate management of high-risk group | Smartphone application providing diagnostic and treatment protocols, with module for control of drugs and supplies | ✘ | ✔ | ✘ | ✘ | ✘ | ✘ | ✔ |

## Table S6. Risk of bias of included studies


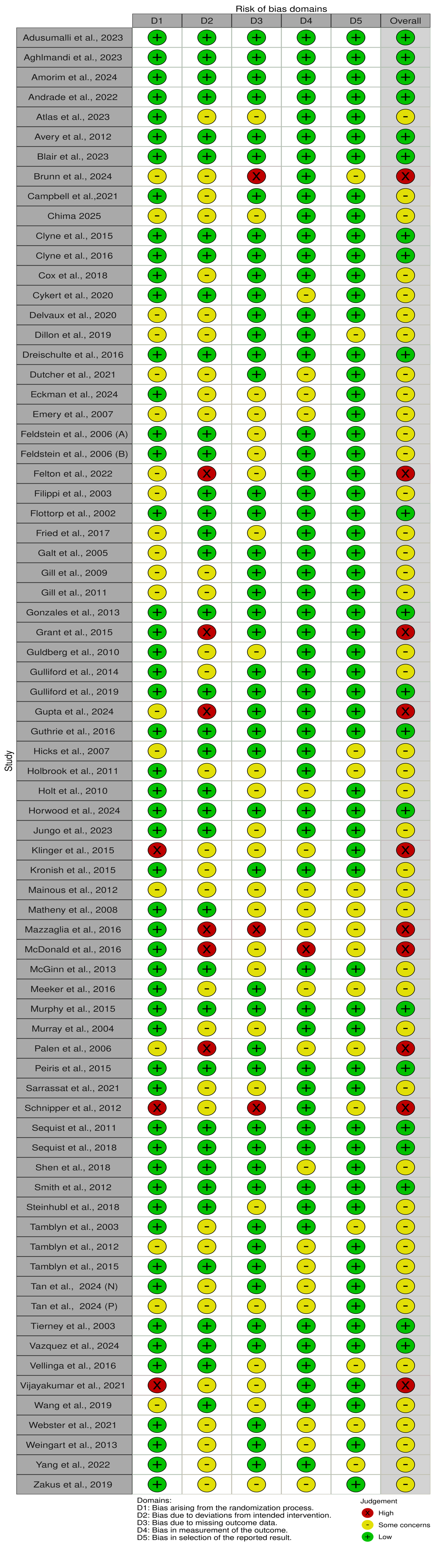


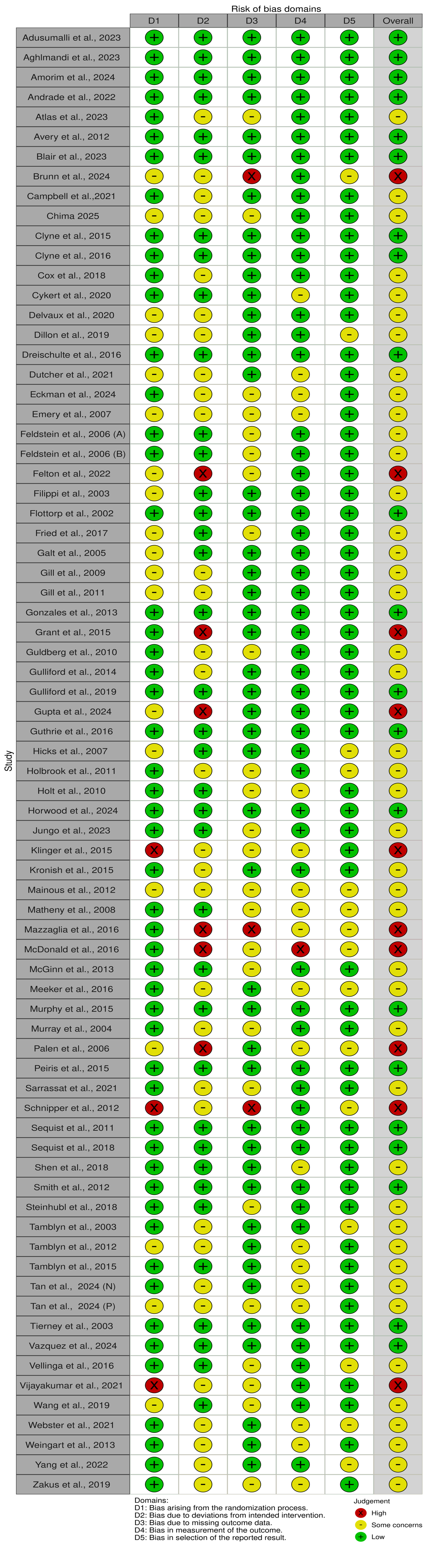


Produced using robvis from: *McGuinness, LA, Higgins, JPT. Risk-of-bias VISualization (robvis): An R package and Shiny web app for visualizing risk-of-bias assessments. Res Syn Meth. 2020; 1- 7. https://doi.org/10.1002/jrsm.1411*

## Figure S1. Visual funnel plot of meta-analysis on medication safety process measures


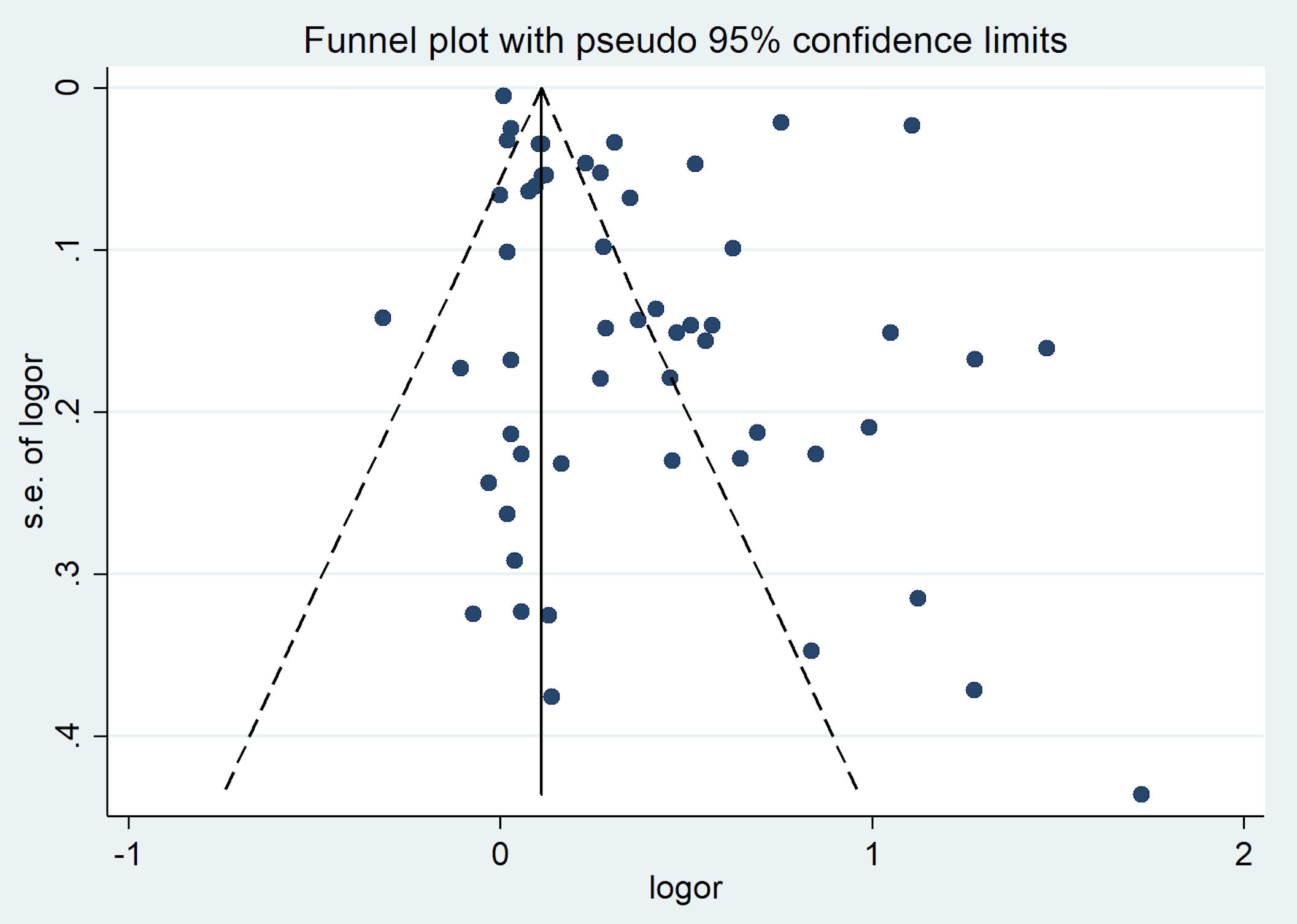


Abbreviations: logor= Log odds ratio, s.e. = standard error.

## Figure S2. Meta-analysis subanalysis on medication safety process measures including only 17 studies at low risk of bias


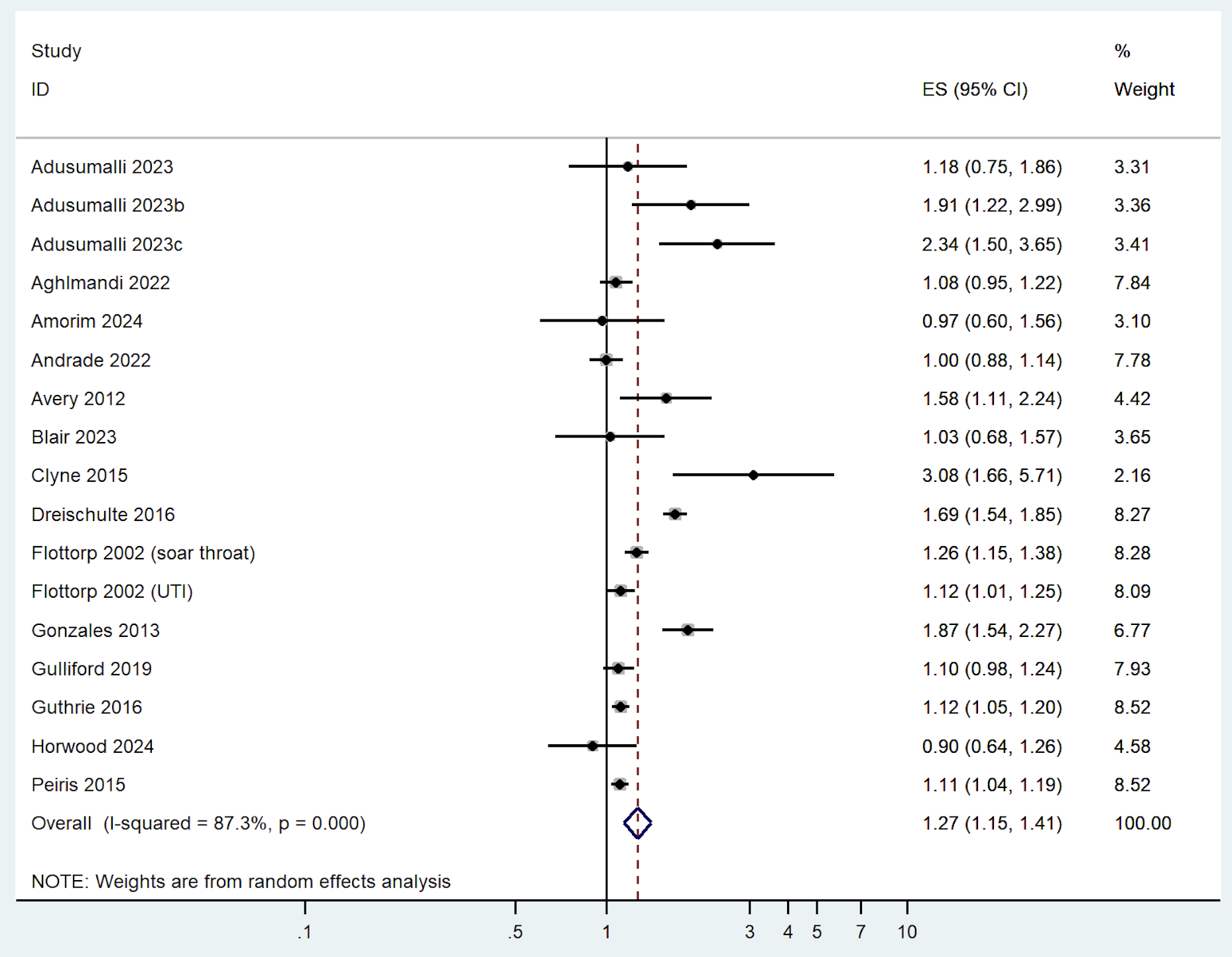


The size of squares is proportional to the weight of each study, with the horizontal lines indicating the 95% confidence interval (CI) of each study; diamond = the pooled estimate with 95% CI; and ES = effect size.

## Figure S3. Meta-analysis results on medication safety process measures comparing interventions including clinical decision support systems, audit and feedback, or both


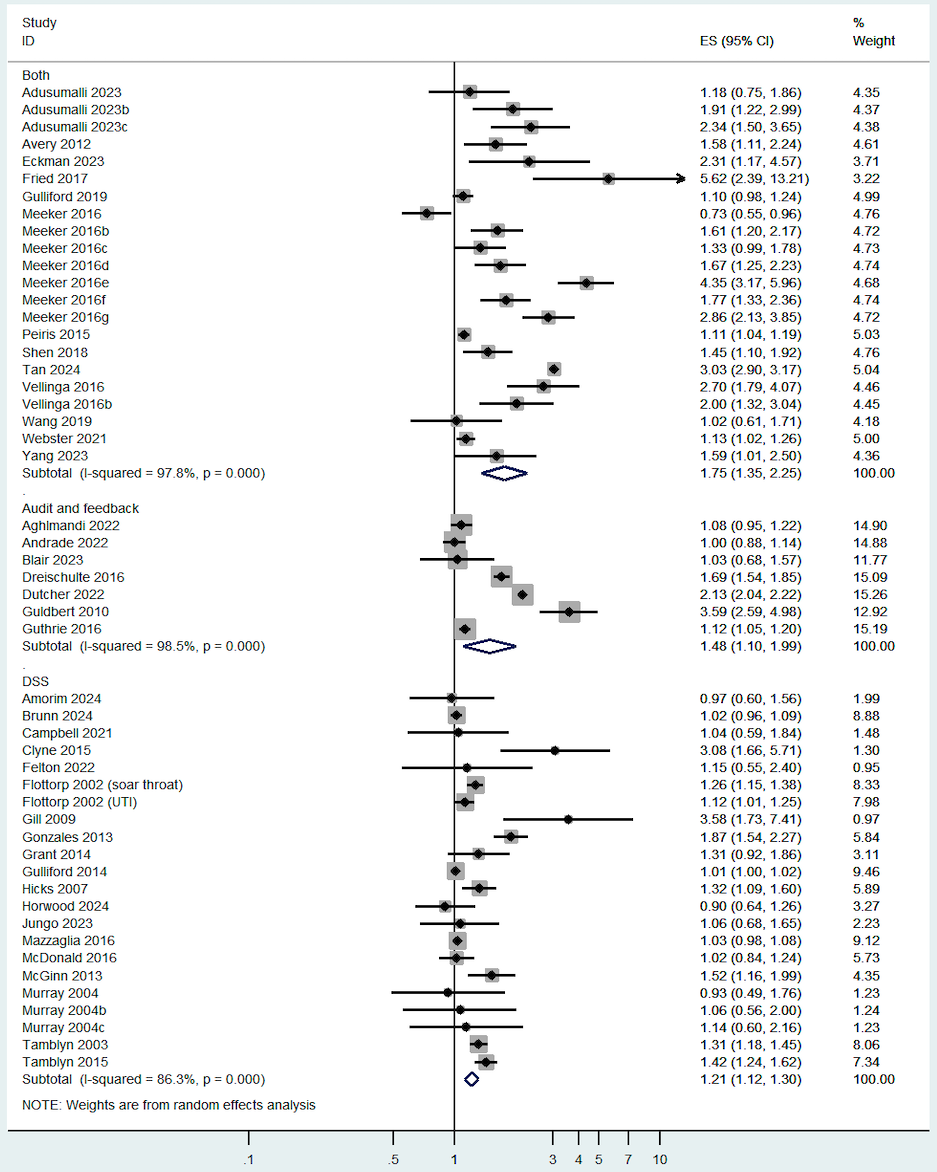


The size of squares is proportional to the weight of each study, with the horizontal lines indicating the 95% confidence interval (CI) of each study; diamond = the pooled estimate with 95% CI; DSS = Decision Support System; and ES = effect size.

## Figure S4. Meta-analysis results on medication safety process measures comparing interventions with or without a patient-facing component


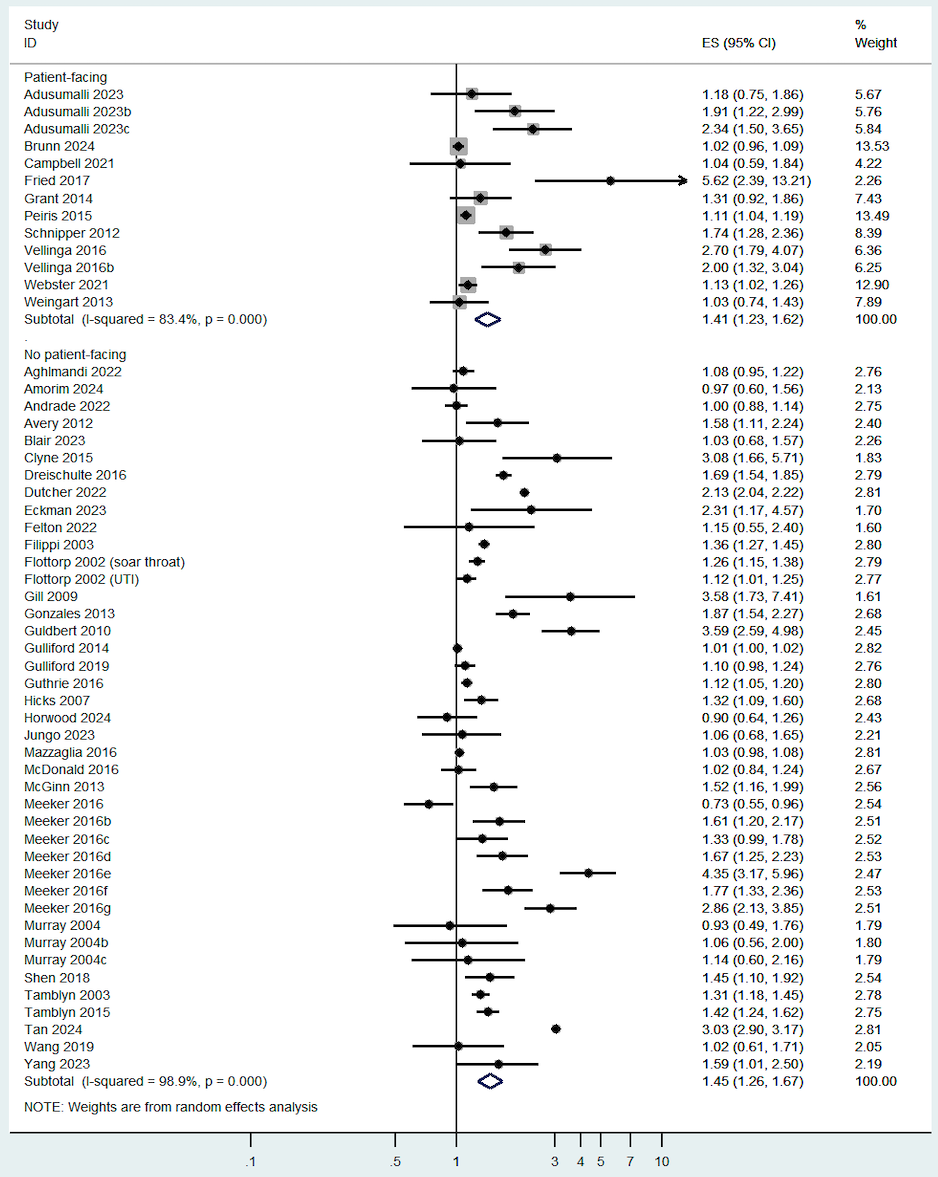


The size of squares is proportional to the weight of each study, with the horizontal lines indicating the 95% confidence interval (CI) of each study; diamond = the pooled estimate with 95% CI; and ES = effect size.

## Figure S5. Visual funnel plot of meta-analysis on non-medication process measures


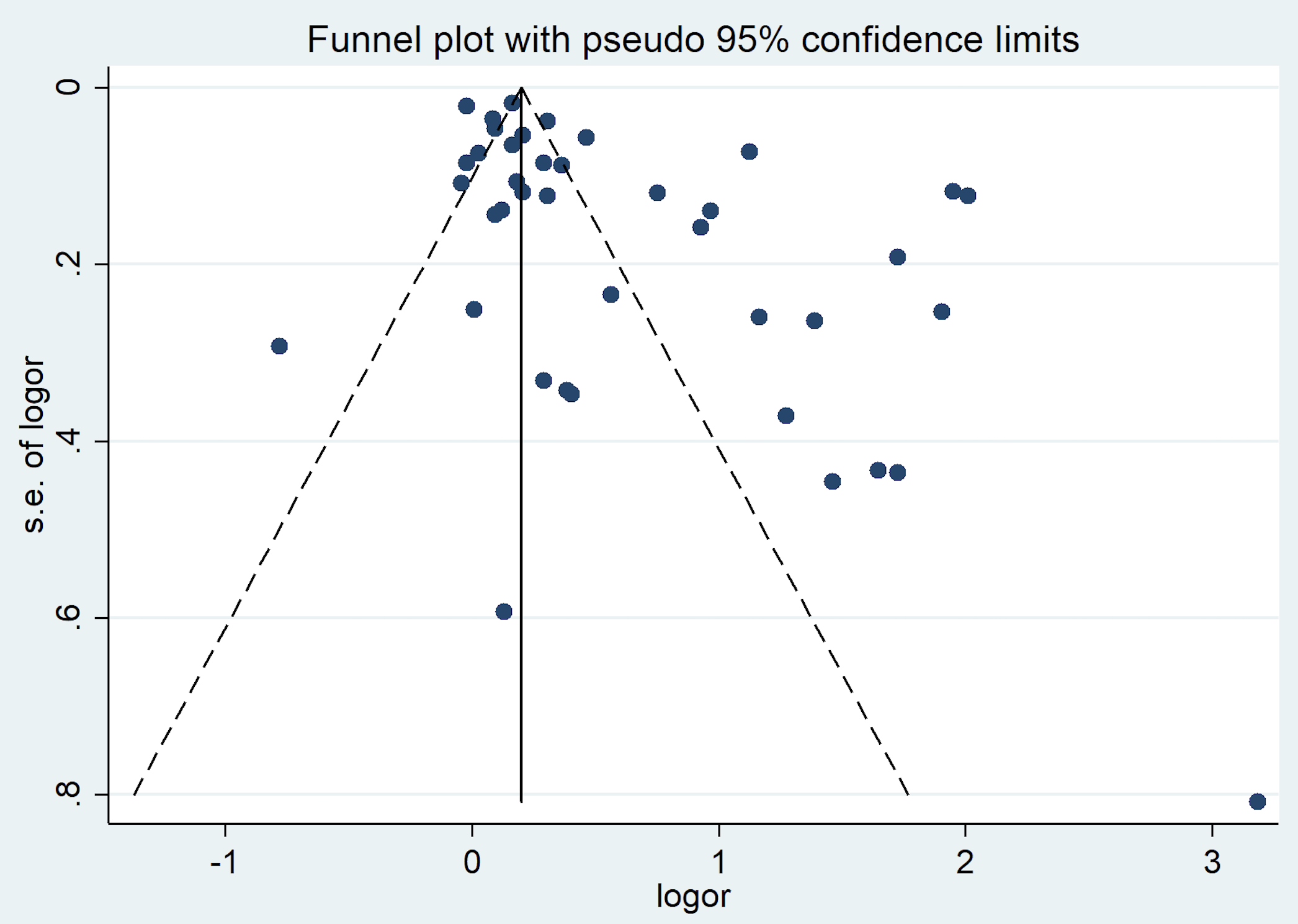


Abbreviations: logor= Log odds ratio, s.e. = standard error.

## Figure S6. Meta-analysis subanalysis on non-medication process measures including only 12 studies at low risk of bias


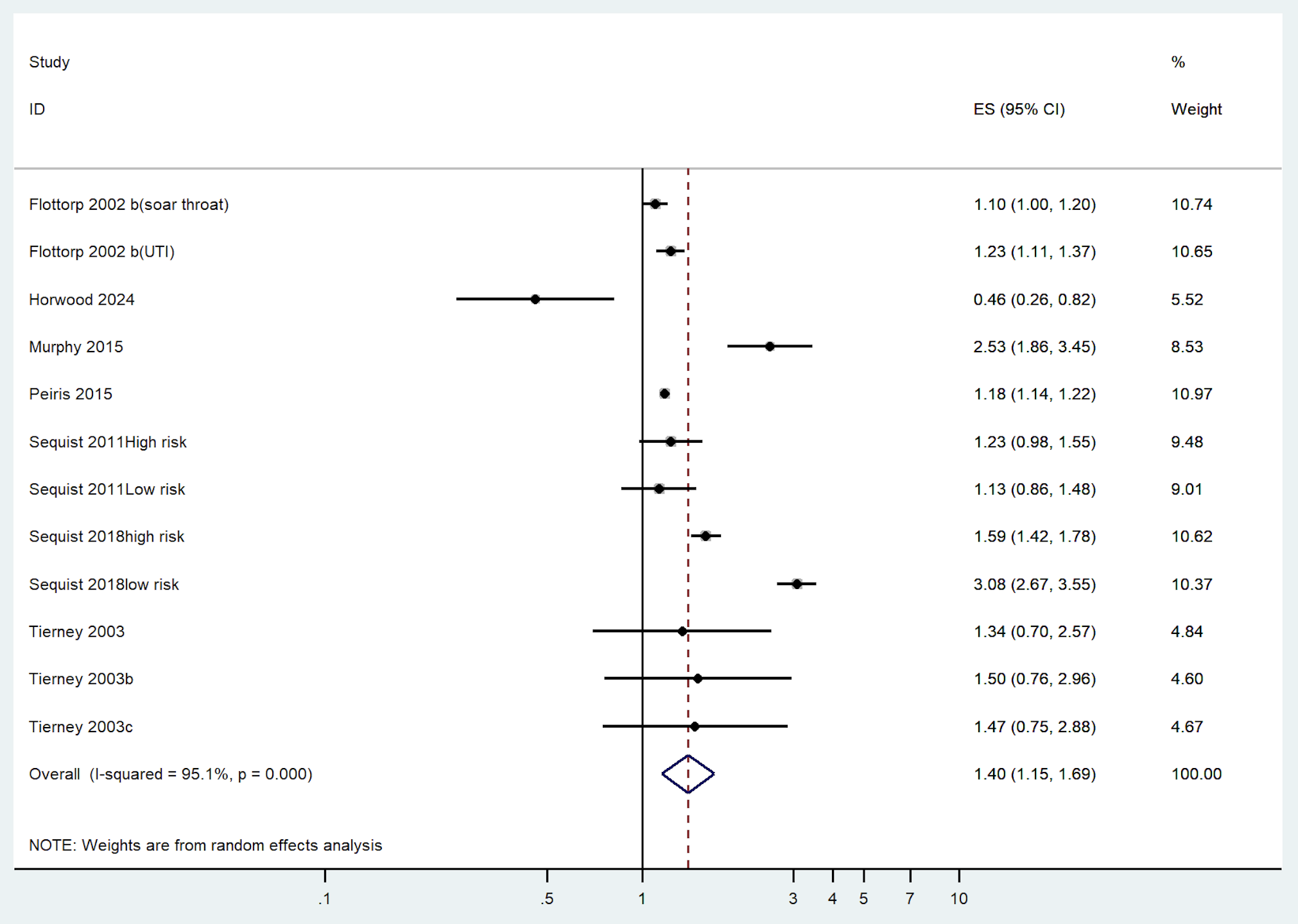


The size of dots is proportional to the weight of each study, with the horizontal lines indicating the 95% confidence interval (CI) of each study; diamond = the pooled estimate with 95% CI; and ES = effect size.

## Figure S7. Meta-analysis results on non-medication process measures comparing interventions including clinical decision support systems, audit and feedback, or both


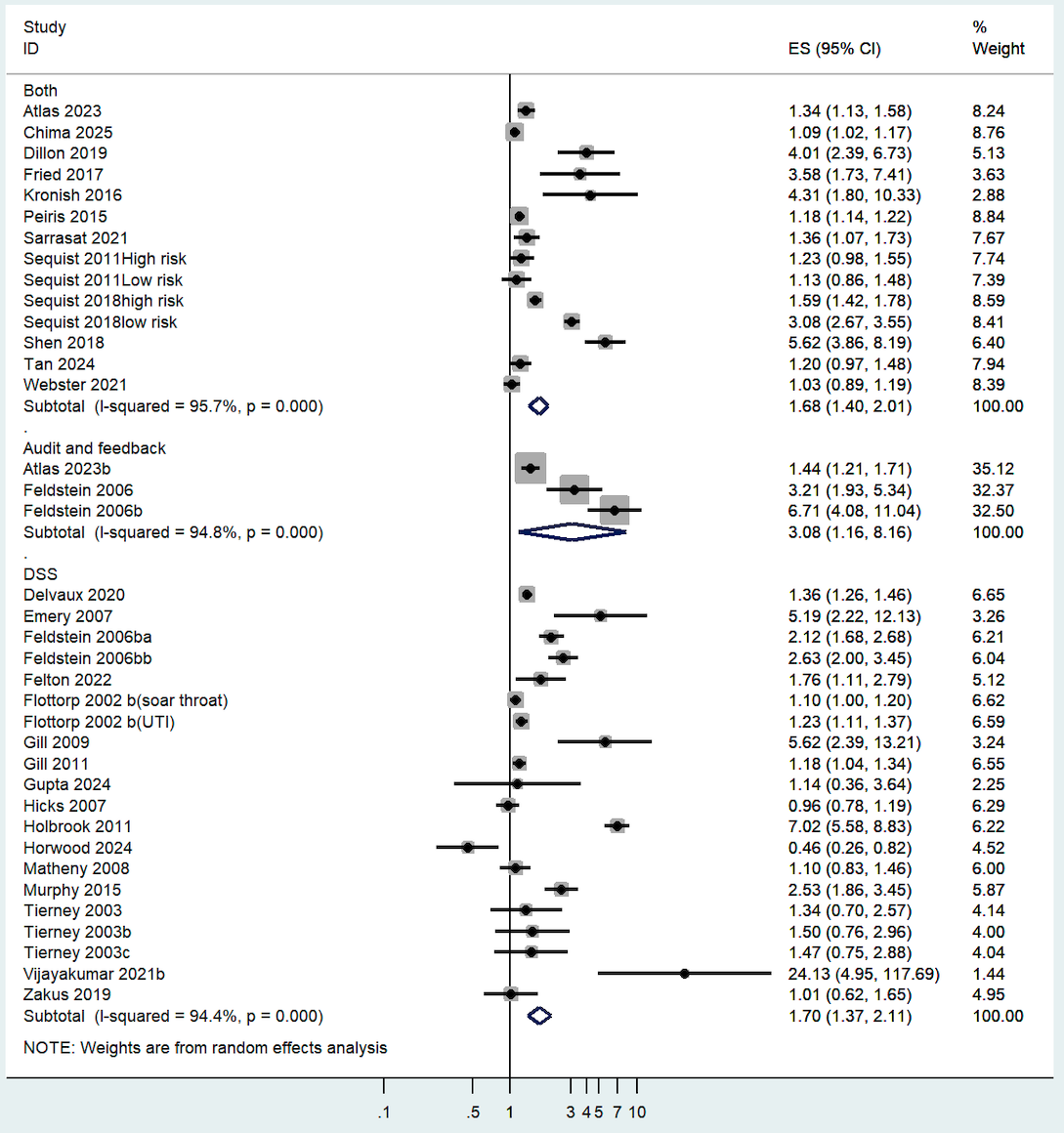


The size of squares is proportional to the weight of each study, with the horizontal lines indicating the 95% confidence interval (CI) of each study; diamond = the pooled estimate with 95% CI; DSS = Decision Support System; and ES = effect size.

## Figure S8. Meta-analysis results on non-medication process measures comparing interventions with or without a patient-facing component


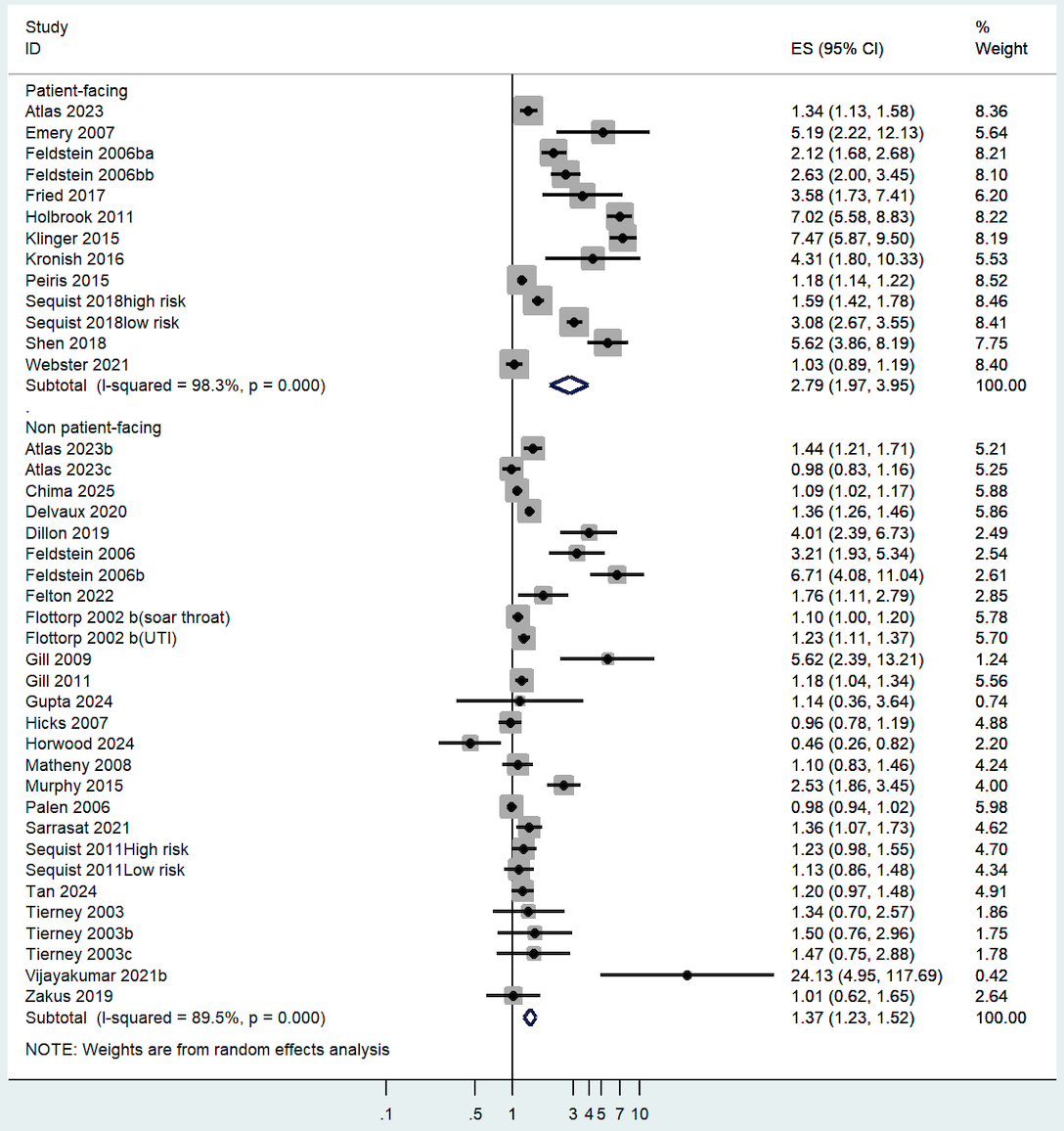


The size of squares is proportional to the weight of each study, with the horizontal lines indicating the 95% confidence interval (CI) of each study; diamond = the pooled estimate with 95% CI; and ES = effect size.

## Figure S9. Visual funnel plot of meta-analysis on adverse events


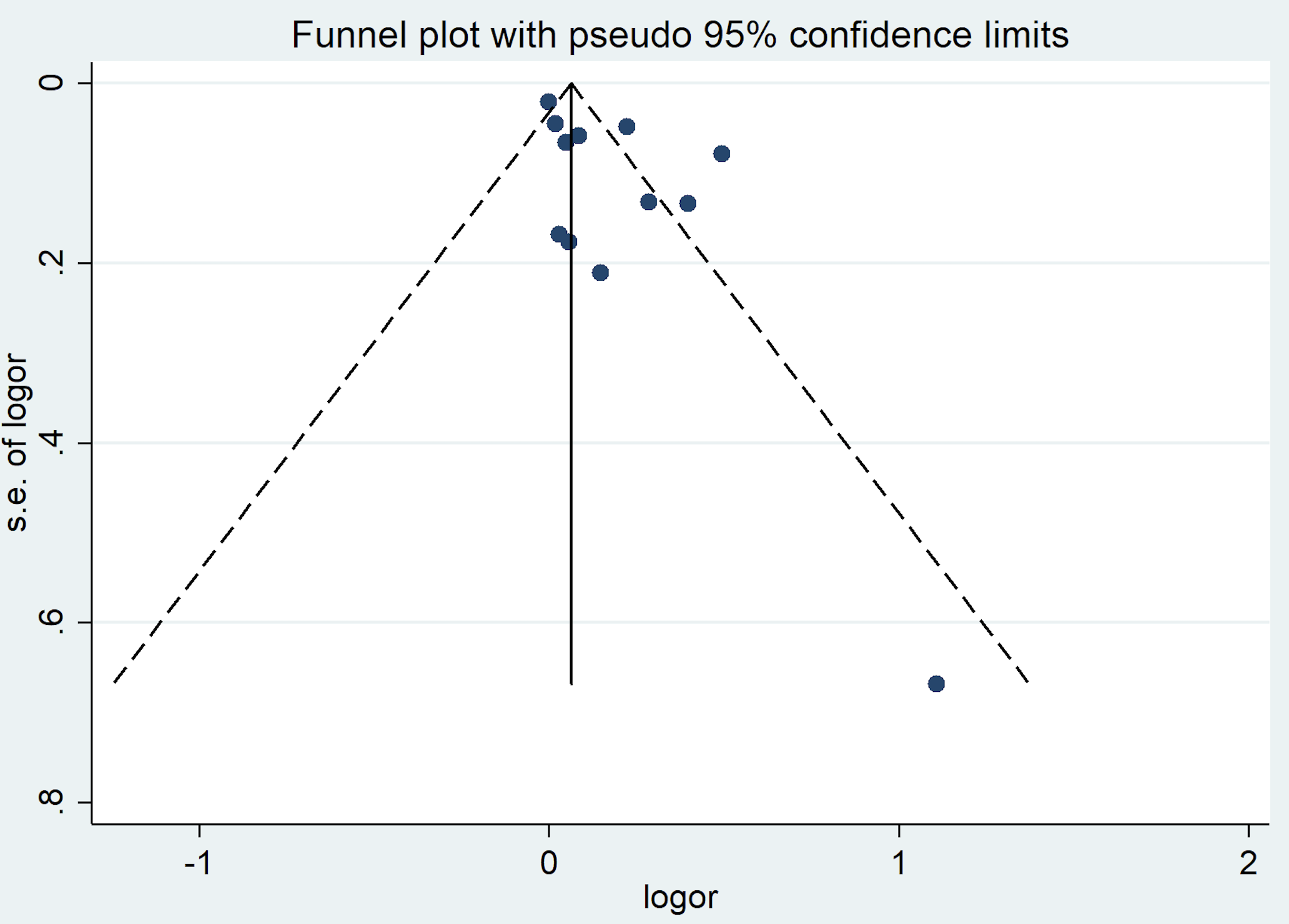


Abbreviations: logor= Log odds ratio, s.e. = standard error.

## Figure S10. Meta-analysis subanalysis on adverse events including only 5 studies at low risk of bias


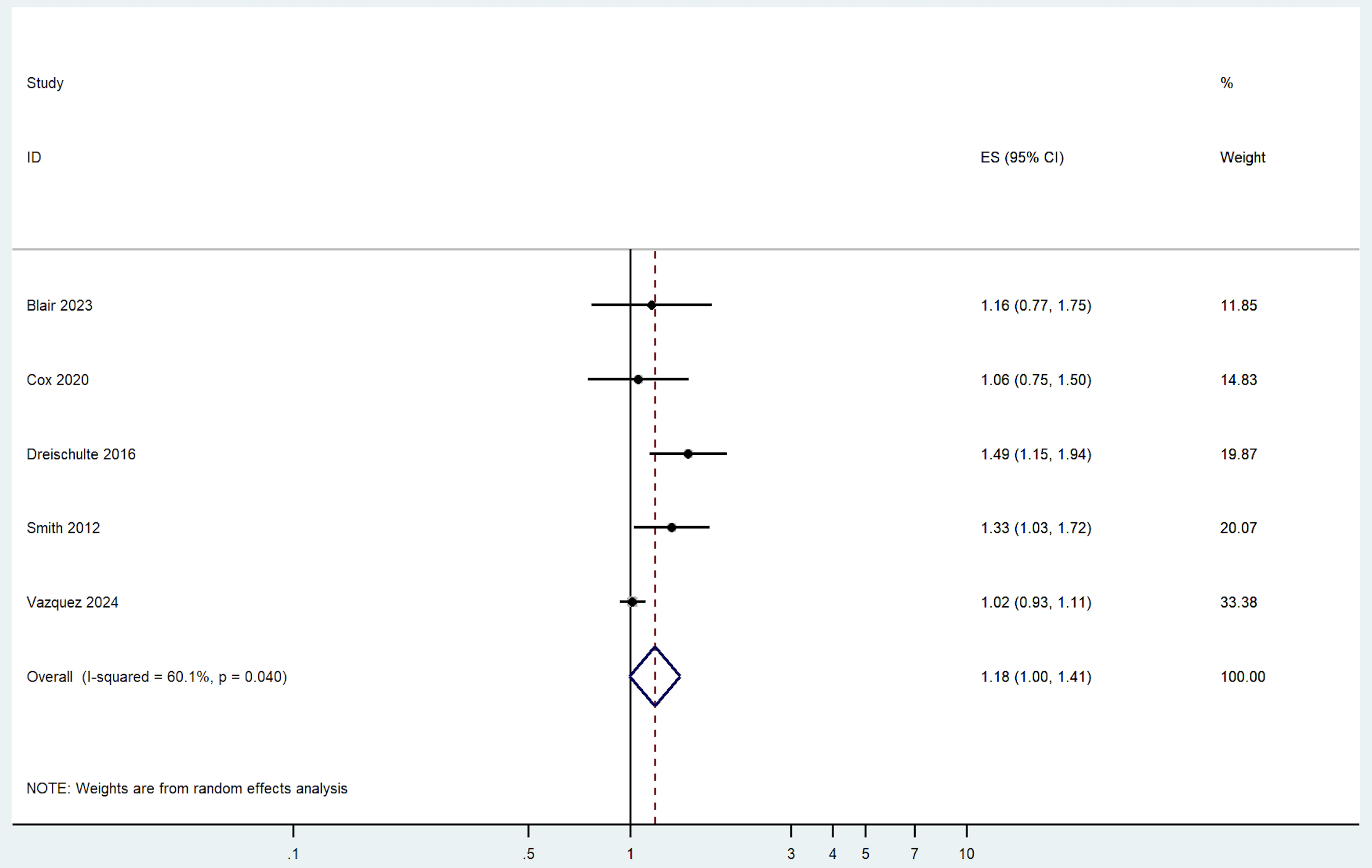


The size of dots is proportional to the weight of each study, with the horizontal lines indicating the 95% confidence interval (CI) of each study; diamond = the pooled estimate with 95% CI; and ES = effect size.
